# 3,4,5‐Trihydroxypiperidine Based Multivalent Glucocerebrosidase (GCase) Enhancers

**DOI:** 10.1002/cbic.202200077

**Published:** 2022-04-07

**Authors:** Costanza Vanni, Francesca Clemente, Paolo Paoli, Amelia Morrone, Camilla Matassini, Andrea Goti, Francesca Cardona

**Affiliations:** ^1^ Dipartimento di Chimica “Ugo Schiff” DICUS Università di Firenze Via della Lastruccia 3–13 50019 Sesto Fiorentino (FI) Italy; ^2^ Dipartimento di Scienze Biomediche Sperimentali e Cliniche Mario Serio Università di Firenze Viale Morgagni 50 50134 Firenze Italy; ^3^ Laboratorio di Biologia Molecolare delle Malattie Neurometaboliche del Centro di Eccellenza di Neuroscience AOU Meyer, Firenze, and Dipartimento di Neuroscienze Area del Farmaco e Salute del Bambino (NEUROFARBA) Università di Firenze Viale Pieraccini n. 24 50139 Firenze Italy; ^4^ Associated with LENS Via N. Carrara 1 50019 Sesto Fiorentino Italy

**Keywords:** Gaucher disease, glucocerebrosidases, glycosidase inhibition, iminosugars, multivalent effect

## Abstract

The synthesis of five new multivalent derivatives of a trihydroxypiperidine iminosugar was accomplished through copper catalyzed alkyne‐azide cycloaddition (CuAAC) reaction of an azido ending piperidine and several propargylated scaffolds. The resulting multivalent architectures were assayed as inhibitors of lysosomal GCase, the defective enzyme in Gaucher disease. The multivalent compounds resulted in much more potent inhibitors than a parent monovalent reference compound, thus showing a good multivalent effect. Biological investigation of these compounds as pharmacological chaperones revealed that the trivalent derivative (**12**) gives a 2‐fold recovery of the GCase activity on Gaucher patient fibroblasts bearing the L444P/L444P mutations responsible for neuropathies. Additionally, a thermal denaturation experiment showed its ability to impart stability to the recombinant enzyme used in therapy.

## Graphical abstract

**The multivalent piperidine iminosugars** described in this work are strong inhibitors of glucocerebrosidase (GCase), the defective enzyme in Gaucher disease. The trivalent derivative **12** was able to double GCase activity in patient's fibroblasts homozygous for the L444P mutation, which is resistant to most pharmacological chaperones. Co‐incubation of **12** with recombinant GCase enzyme paves the way to its application in PC/ERT therapy.

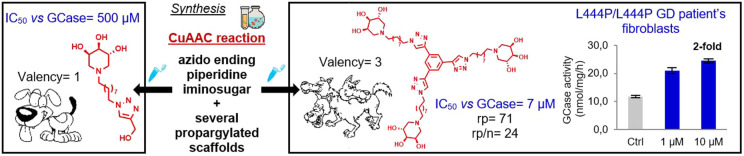

## Introduction

The concept of multivalency in the field of enzyme inhibition and its biological consequences, particularly with glycosidases, has become a “hot topic” research, following the first example of a trivalent deoxynojirimycin (DNJ) derivative showing a small but quantifiable inhibitory multivalent effect (MVE) towards Jack bean α‐mannosidase in 2009.^[1]^

The MVE, previously well‐known in the field of carbohydrate‐lectin interactions,^[2]^ can be defined, in the context of enzyme inhibition, as the increase in the inhibitory activity observed for compounds having more than one bioactive unit (inhitope) linked to a scaffold compared to the individual units. Since the simultaneous presentation of multiple binding units may increase the relative potency (rp) of the multivalent inhibitor simply due to an increase in local concentration in proximity of the active site, Winum, Ulrich and co‐workers proposed a quantitative assessment of this phenomenon. In particular, they defined the MVE as the ratio between the relative potency (rp) of the multivalent inhibitor and the number of binding units n (i. e. rp/n), and establishing a *positive* MVE when this ratio is higher than 1.^[3]^ Only in this case, indeed, there is a real advantage of using a multivalent inhibitor (which often requires lengthy synthesis), since the same inhibitory potency cannot be achieved by increasing the concentration of the monovalent compound.^[4]^

The MVE in the context of glycosidase inhibition has been studied with iminosugars^[5]^ as the bioactive inhitopes and a plethora of different scaffolds.[^6^, ^7^]

However, most of the reported examples concern the multimerization of DNJ and its effect on the inhibition of commercially available Jack bean α‐mannosidase, while the effect of multivalent ligands on therapeutically relevant enzymes is much less investigated.

In this context, and to partially fill this gap, we recently reported that the multimerization of the natural compound 1,4‐dideoxy‐1,4‐imino‐d‐arabinitol (DAB‐1) resulted in a relevant MVE towards the lysosomal enzyme *N*‐acetylgalactosamine‐6‐sulfatase (GALNS), involved in the Mucopolysaccharidosis IVA or Morquio A.^[8]^

Other multimeric pyrrolidine iminosugars showed a remarkable MVE towards human α‐galactosidase A (α‐Gal A), an enzyme involved in Fabry disease, and behave as enzyme enhancers when tested on cell lines.^[9]^

It was also reported that the multimerization of polyhydroxylated acetamidoazepanes improved the inhibitory potency and the selectivity profile towards relevant human and bacterial hexosaminidase.^[10]^ Golgi α‐mannosidase (GMIIb) from *Drosophila melanogaster*, a model target enzyme for anticancer therapy, was also reported to be strongly inhibited by a porphyrin‐based DNJ derivative^[11]^ and by dendrimer and resorcinarene based DAB‐1 architectures.^[12]^

The role of lysosomal acid β‐glucosidase (glucocerebrosidase, also known as GCase, EC 3.2.1.45, MIM*606463) in determining the onset of Gaucher disease (GD), the most common among lysosomal storage disorders (LSDs), is known since long time. GD is caused by mutations in the *GBA* gene (mapped on chromosome: 1q21‐22), which encodes for GCase, deputed to the hydrolysis of glucosylceramide (GlcCer). Reduced or absent GCase activity leads to accumulation of GlcCer in the lysosomes with the consequent clinical symptoms of GD starting from mild to neuronopathic severe form.^[13]^

Much more recent findings have suggested the involvement of GCase in the onset of Parkinson's disease (PD), the second most common neurodegenerative disorder. Among the known genetic risk factors for PD, mutations in *GBA* are the most common.^[14]^

The molecular basis connecting GD to PD well explains why the modulation of GCase activity is emerging as a key therapeutic target for both pathologies.^[15]^

Among glycomimetics, iminosugars are attractive potential therapeutics towards GD and other LSDs in the emerging so‐called pharmacological chaperone therapy (PTC). This approach derives from the observation of the counter‐intuitive effect of glycosidases inhibitors in enhancing the enzyme activity, thus acting as chaperones, when they are employed at sub‐inhibitory concentration.

PCs favor the mutated enzyme correct folding in the endoplasmic reticulum (ER), thus facilitating its trafficking to the lysosomes, where the chaperone is replaced by the natural substrate. This mechanism allows the enzyme to recover some hydrolytic activity, compromised as a consequence of the genetic mutations.^[16]^

The identification of new inhibitors of GCase is of paramount importance for finding new PCs for this enzyme, since there are no PCs yet on the market for the treatment of GD or PD. Moreover, considering that the most common treatment available for GD is the enzyme replacement therapy (ERT) with recombinant enzyme (imiglucerase, taliglucerase‐α or velaglucerase‐α), finding new ligands able to stabilize the enzyme used for therapy may have a great impact in a combined PC‐ERT therapy (which was investigated for Fabry disease, another LSD)^[17]^ to reduce patients’ hospitalization and the side effects and cost of ERT.^[18]^

To our knowledge, multivalent inhibitors of GCase have not been reported yet, apart from a single example by Compain and co‐workers, who found a small but significant MVE in GCase inhibition for two DNJ‐based clusters, which were also able to enhance enzyme activity in cell lines.^[19]^

During our studies in the synthesis of new inhibitors/chaperones for GCase, we identified derivatives of a trihydroxypiperidine, enantiomer of a natural product, as good inhitopes for the enzyme, as long as they possess an alkyl chain with at least eight carbon atoms linked to the nitrogen or to the adjacent carbon atom.^[20]^

Accordingly, preliminary studies aimed at the multimerization of the trihydroxypiperidine with a shorter linker (*i. e*., three carbon atoms)^[21]^ resulted only in a very modest inhibition of GCase for a nonavalent derivative (22 % inhibition at 1 mM, data unpublished).

In this work, we report the synthesis of a series of new multivalent trihydroxypiperidines bearing a C9‐linker at the nitrogen atom and their biological evaluation towards human GCase. The chaperoning properties of the best inhibitor have also been investigated, as well as its ability to inhibit/stabilize the recombinant enzyme used for therapy.

## Results and Discussion

Piperidine **2** was synthesized from the d‐mannose derived aldehyde **1** as previously reported.^[22]^

**Scheme 1:**
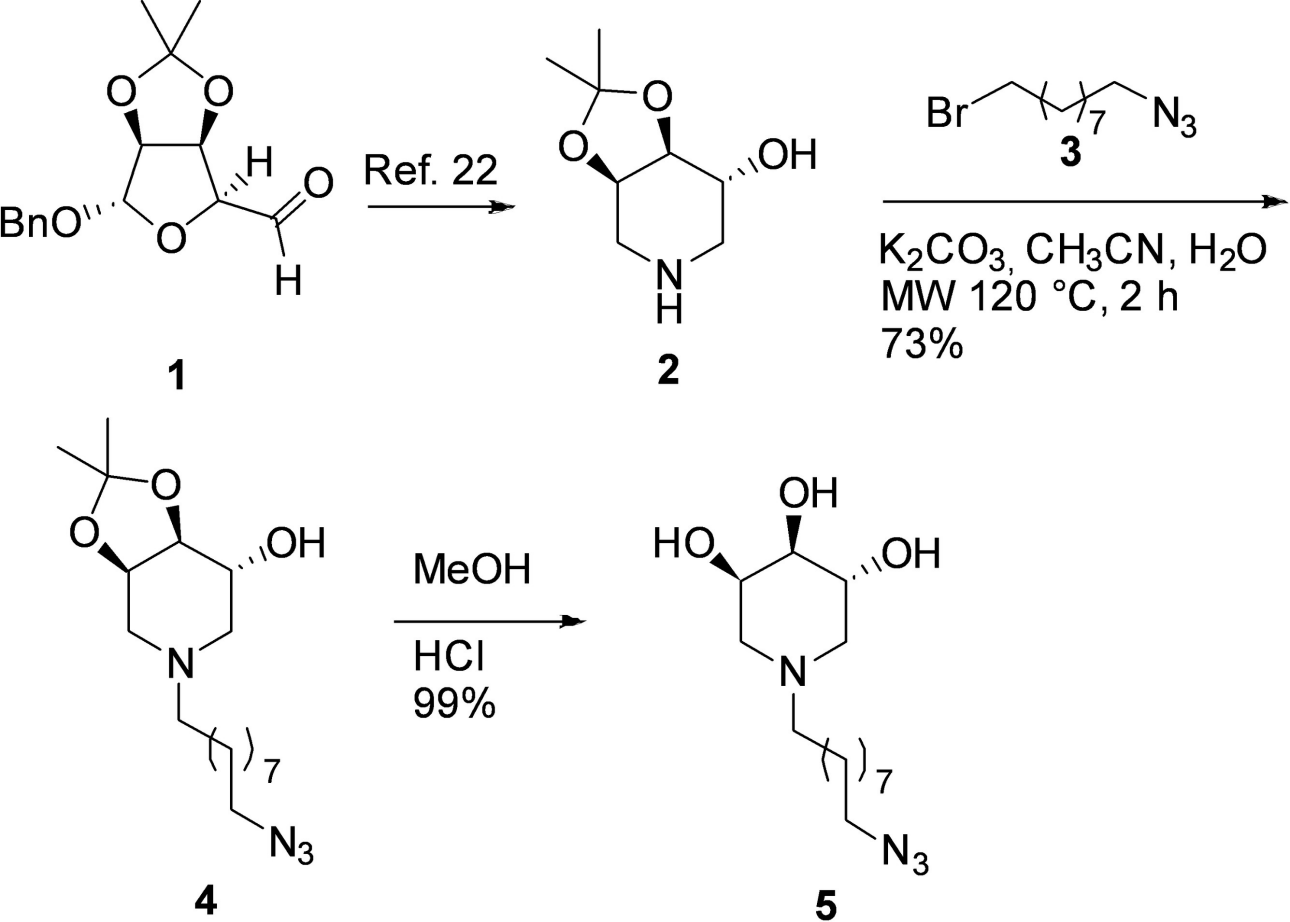
Synthesis of the trihydroxypiperidine **5**.

With the piperidine active motif in hand, the desired linker was appended by *N*‐functionalization with a proper azido‐ending bromo derivative **3** bearing nine carbon atoms, achieved by heating at 120 °C under microwave irradiation in the presence of potassium carbonate as the base. These conditions gave the protected piperidine **4** (73 %), which was then deprotected under acidic conditions (aq. HCl in MeOH), providing the azido‐ending trihydroxypiperidine **5** in almost quantitative yield (Scheme 1).

**Figure 1:**
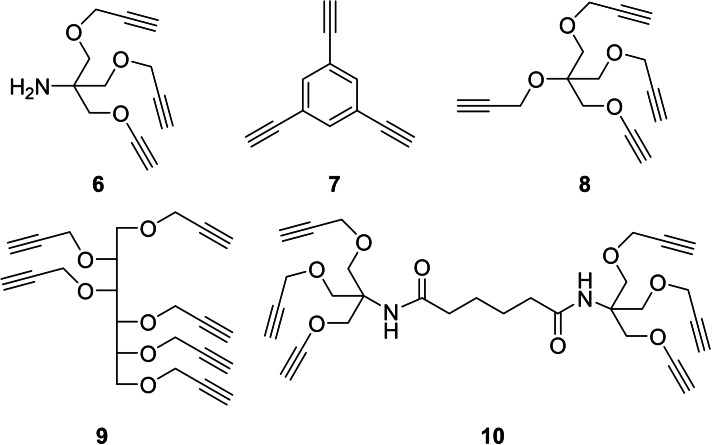
Multivalent alkyne scaffolds employed in this work.

Compound **5** was then reacted through the copper catalyzed azide‐alkyne cycloaddition (CuAAC) reaction,^[23]^ one of the most versatile reaction for the synthesis of multivalent ligands, with a series of propargylated scaffolds with different valencies and topologies, shown in Figure 1. We chose to employ the deprotected **5** (and not the protected **4**) in the CuAAC reactions, since we previously encountered several problems in the purification of the multivalent architectures after acetonide removal under acidic conditions, due to the basicity of the multivalent piperidine iminosugars.[^8a^, ^21^]

The trivalent scaffold 1,3,5‐triethynylbenzene (**7**) is commercially available and was directly employed as such, while the other scaffolds shown in Figure 1 were synthesized through propargylation of the corresponding alcohols with propargyl bromide and NaH or through straightforward multistep procedures reported previously. In particular, the tetravalent **8** was prepared through propargylation of pentaerythritol,^[24]^ and the hexavalent scaffold **9** through propargylation of d‐mannitol.^[25]^

The trivalent tris[(propargyloxy)methyl]aminomethane (**6**) was prepared in a three‐step sequence from tris (hydroxymethyl) aminomethane as previously reported,^[26]^ and the hexavalent **10** was synthesized starting from adipic acid and **6**.[^9^, ^27^]

The CuAAC reactions were performed with a slight equivalent excess of **5** per alkyne moiety reported in Figure 1, in presence of a catalytic amount of CuSO_4_ and sodium ascorbate in a 2 : 1 THF/H_2_O mixture under MW irradiation at 80 °C for 45 minutes. These conditions, in our hands, gave the best yields of the corresponding multivalent iminosugars.[^12b^, ^21^, ^28^]

Copper complexation by the triazole rings was avoided thanks to a treatment with a copper‐scavenger resin (QuadraSil^®^MP) after the CuAAC reaction, followed by further purification of the multivalent architectures through size exclusion chromatography (SEC) with Sephadex LH‐20 using water as a solvent.

**Scheme 2:**
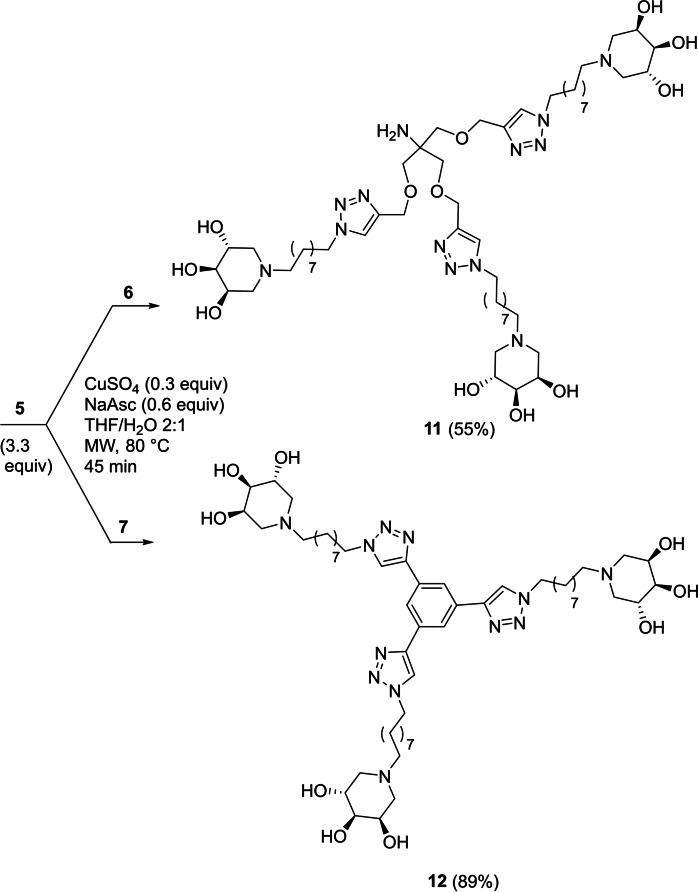
Synthesis of the trivalent iminosugars **11** and **12**.

Following this procedure, the two trivalent derivatives **11** and **12** were obtained in 55 % and 89 % yields by reaction of tris[(propargyloxy)methyl]aminomethane (**6**), or commercially available **7**, respectively, with 3.3 molar equivalents of **5** (Scheme 2).

**Scheme 3:**
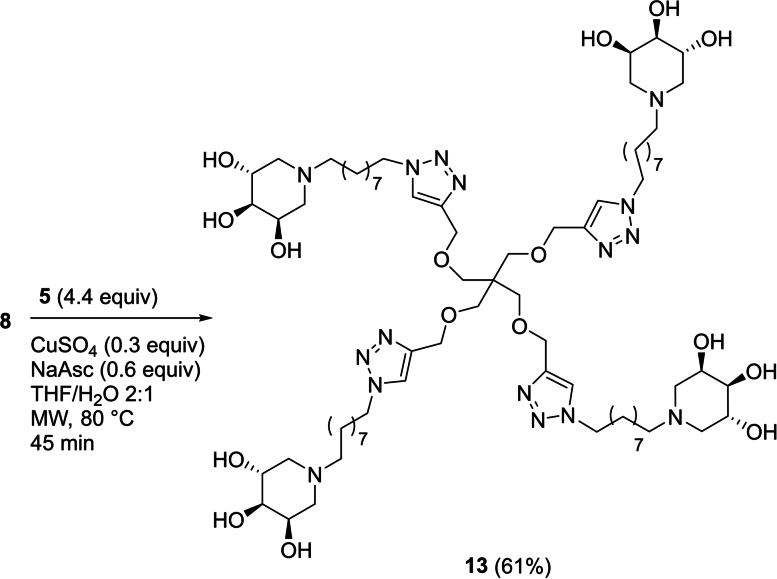
Synthesis of tetravalent iminosugar **13**.

The tetravalent compound **13** was obtained in 61 % yield through reaction of the scaffold **8** with 4.4 equiv. of **5** (Scheme 3).

**Scheme 4:**
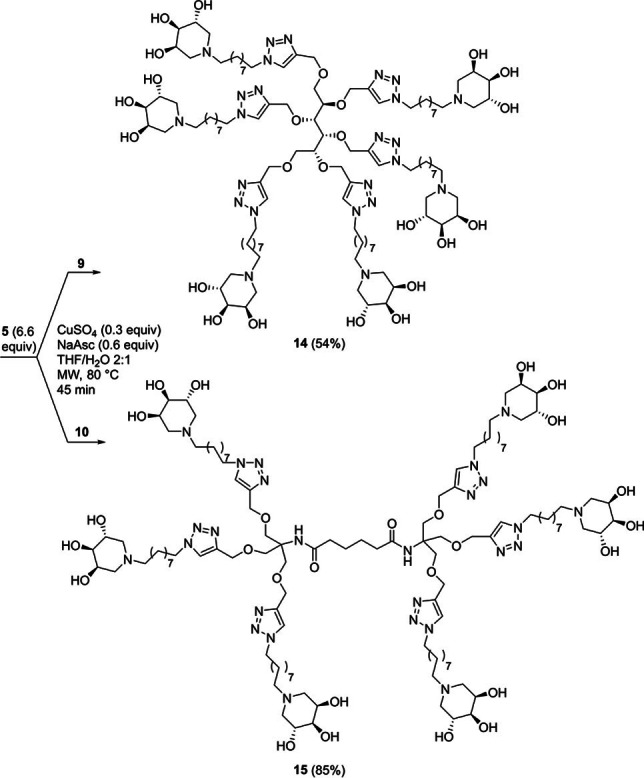
Synthesis of the hexavalent iminosugars **14** and **15**.

The two hexavalent derivatives **14** and **15** were obtained in 54 % and 85 % yields, respectively, through reaction of the scaffolds **9** and **10** with 6.6 equiv. of the piperidine **5** (Scheme 4).

**Scheme 5:**
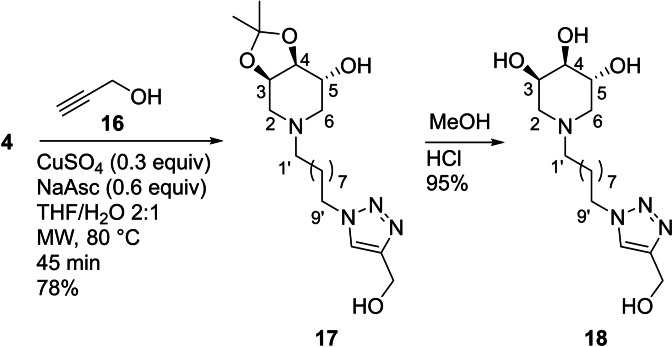
Synthesis of the monovalent reference compound 1**8**.

To evaluate the relative inhibitory activity enhancement of these new multivalent architectures, a proper monovalent counterpart was also synthesized. Starting from the protected azido derivative **4**, the CuAAC reaction was performed with propargyl alcohol (**16**), in the presence of CuSO_4_/sodium ascorbate in THF/H_2_O=2 : 1 at 80 °C for 45 minutes, affording the adduct **17** in 78 % yield (Scheme 5). Deprotection with MeOH/HCl and treatment with strongly basic resin Ambersep 900‐OH afforded the monovalent compound **18** in 95 % yield.

Preliminary biological evaluation of all the multivalent piperidines was carried out by measuring their inhibitory activity towards human GCase. The compounds were screened in extracts from a pool of human leucocytes isolated from healthy donors (1 mM inhibitor concentration, 37 °C and optimal pH conditions, see Experimental Section). The results are summarized in Table 1, where the inhibition of the monovalent piperidine **18** is also reported in order to evaluate the rp and rp/n values. Compound **18** displayed ca. 70 % inhibition at 1 mM inhibitor concentration, while all the new multivalent trihydroxypiperidines **11–15** inhibited GCase more strongly (80‐100 %), showing that the multivalent presentation leads to an enhancement of the biological activity. Indeed, the IC_50_ calculated for compound **18** (500 μM) was more than one order of magnitude higher than those calculated for the multivalent compounds **11–15** (6‐27 μM). The relative potency (rp) of compounds **11–15** ranged from 19 for the trivalent derivative **11** to 83 for the hexavalent derivative **14**, demonstrating that the multivalent compounds are much more potent inhibitors than the monovalent one. The best inhibitor of the series was compound **14**, which showed an IC_50_ of 6 μM and a relative potency of 83 with respect to the monovalent counterpart. In all cases the rp/n values were higher than 1, showing the existence of a *positive multivalent effect*.^[3]^ In particular, the best rp/n value was shown for the trivalent compound **12**, which has an IC_50_ value close to that of **14** (7 μM) but only three inhitope binding units (rp/n=24). When compared to compound **11** with the same valency and rp/n=6, it appears that the presentation of the binding units in compound **12** is beneficial for the inhibitory activity. The different topological orientation of the inhitopes in the multivalent architecture was also relevant in the case of the two hexavalent compounds **14** and **15**, which showed quite different rp/n.

**Table 1:** Inhibitory activity of the trihydroxypiperidines **11–15** and **18**.

Compound	Valency	GCase inhibition^[a]^	IC_50_ [μM]^[b]^	rp	rp/n
**18**	1	69	500±50	–	–
**11**	3	100	27±3	19	6
**12**	3	100	7±1	71	24
**13**	4	100	9±4	56	14
**14**	6	80	6±2	83	14
**15**	6	100	11±3	45	8

[a] Percentage inhibition of GCase in human leukocytes extracts incubated with the inhibitor (1 mM). [b] IC_50_ values were determined by measuring GCase activity at different concentrations of each inhibitor.

Kinetic investigation on compound **12** revealed a pure competitive inhibition, with a calculated K_*i*_=3.1±0.2 μM (see the Supporting Information file).

**Figure 2:**
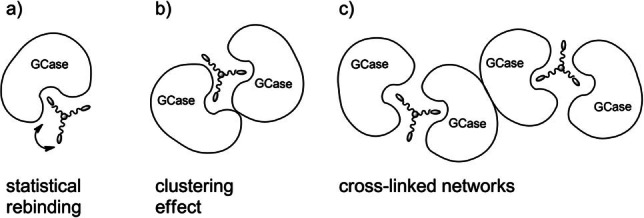
Cartoon of the possible interaction of compound **12** with the GCase enzyme.

GCase is reported to be inhibited by multivalent inhibitors^[19]^ although it has a single and buried active site as many other glycosidases. Therefore, the multivalent effect observed for compound **12** might be ascribed to a statistical rebinding mode^[4]^ (Figure 2a). However, the highest rp/n obtained with compound **12** could be also ascribed to a clustering effect (Figure 2b) or a cross‐linked network (Figure 2c), based on some recent reports suggesting GCase dimerization in solution.^[29]^

Contrary to the many reports with other multimeric glycosidases (e. g. Jack Bean Mannosidase) and multivalent ligands, studies with GCase are still in their infancy. Therefore, a rationale of the optimal topological presentation of the inhitopes to maximize the inhibitory effect of the multivalent ligands is still lacking.

Pursuing our goal to discover new GCase enhancers on cell lines bearing Gaucher mutations, three inhibitors with different valencies, namely the trivalent **12**, the tetravalent **13** and the hexavalent **15**, were evaluated as pharmacological chaperones (PCs) towards human fibroblasts derived from Gaucher patients bearing a selected *GBA* mutation (N370S/RecNcil). The results are shown in Table 2 (see the Supporting Information file for more detailed graphs). Fibroblasts derived from GD patients bearing the N370/RecNcil mutations were incubated without (control, ctrl) or with the compounds at six increasing concentrations. After 4 days, the GCase activity was determined in lysates from treated fibroblasts. All the three compounds showed a moderate increase in GCase activity (20‐25 % activity enhancement). This result was encouraging, since a previously synthesized monovalent trihydroxypiperidine bearing a C8 alkyl chain at the nitrogen atom increased the enzyme activity of almost the same value (1.25‐fold) but at a remarkably higher concentration (100 μM).^[20a]^

**Table 2:** Chaperoning activity assays of compounds **12**, **13** and **15** on N370S/RecNcil human fibroblasts.

Compound	**12**	**13**	**15**
GCase activity rescue^[a]^	1.21 at 10 μM	1.26 at 10 μM	1.21 at 50 μM

[a] The best enhancement observed for each compound is reported as the ratio between the activity in the presence of a given concentration and the control.

Given the best rp/n measured for compound **12**, and the commercial availability of the scaffold employed for its synthesis, we focused further biological assessment on this compound. The trivalent **12** was assayed on human fibroblasts bearing the L444P/L444P mutation, which is the most common mutation leading to a severe Gaucher disease phenotype with central nervous system involvement,^[30]^ but is refractory to most pharmacological chaperone candidates.^[16c]^

**Figure 3:**
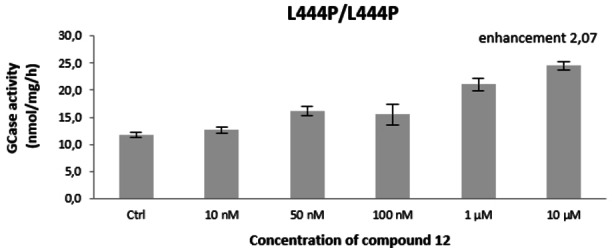
Evaluation of compound **12** as GCase enhancer in fibroblasts derived from GD patients bearing the L444P/L444P mutation. Data for control are obtained as above except that no inhibitor is present (Ctrl).

To our delight, compound **12** gave a remarkable 2‐fold enhancement of GCase activity on these cell lines at 10 μM concentration and was already considerably effective at 1 μM (Figure 3). This enhancement, to the best of our knowledge, is one of the highest ever observed for a PC towards these cell lines.^[31]^

**Figure 4:**
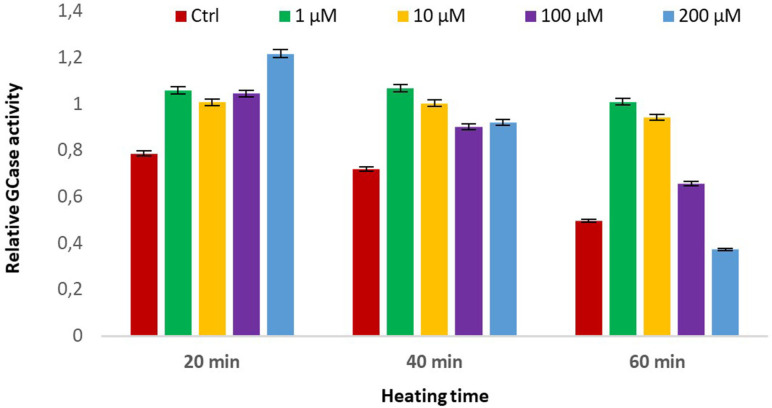
Stabilization of recombinant human GCase using heat inactivation in the presence of compound **12**. Relative enzymatic activity after thermal denaturation (48 °C) for 20, 40 or 60 minutes at the indicated inhibitor concentrations with respect to the corresponding assay at 37 °C. Data for control are obtained as above except that no inhibitor is present (Ctrl).

In order to mimic and estimate the stabilization effect triggered as a PC on misfolded enzymes in cells, compound **12** was tested in a thermal denaturation experiment using recombinant GCase (VPRIV®), the enzyme used in the ERT therapy for the treatment of some forms of Gaucher disease. IC_50_ value resembling the one towards GCase from leukocyte homogenate (4.0±0.4 μM *vs* 7.0±1.0 μM; see the Supporting Information file for more detailed graphs) was obtained. Recovery of recombinant GCase activity was measured at 48 °C in the presence and in the absence (control, ctrl) of increasing concentrations of compound **12** at different incubation times (Figure 4). The trivalent derivative **12** showed a relative stabilization of GCase at all the tested concentrations. Remarkably, the highest relative stabilization effect was observed at the lowest concentration of PC (1 μM), which is consistent with the compound acting as an inhibitor at higher concentrations. These data undoubtedly suggest that compound **12** is also a good candidate as a stabilizer of the recombinant enzyme used for therapy (PC/ERT therapy).

## Conclusion

In conclusion, we report the synthesis of new multivalent compounds (tri‐, tetra‐ and hexavalent iminosugars) by CuAAC reaction of an azido ending trihydroxypiperidine with several propargylated scaffolds. The compounds were designed to bind the GCase enzyme, which is defective in Gaucher disease, and to investigate the biological response to various valencies and topologies.

Biological assays in leucocytes from healthy donors showed a strong multivalent effect in GCase inhibition and highlighted compound **12** as the more promising for further biological investigation. While pharmacological chaperoning on N370S/RecNCil GD patient fibroblasts showed only a moderate chaperoning activity, a remarkable 2‐fold recovery of enzyme activity was measured on L444P/L444P GD patient fibroblasts, which have been shown unresponsive to most PCs. Moreover, a thermal denaturation experiment on the recombinant enzyme used for therapy showed that compound **12** is able to induce resistance to the thermal denaturation at low concentration (1 μM), highlighting this trivalent derivative as a good candidate for further development.

Further studies are ongoing in our laboratories to better elucidate the high activity of compound **12** from a molecular recognition perspective.

## Experimental Section

### Synthesis and characterization of compounds

**General procedures**: Commercial reagents were used as received. All reactions were carried out under magnetic stirring and monitored by TLC on 0.25 mm silica gel plates (Merck F254). Column chromatographies were carried out on Silica Gel 60 (32‐63 μm) or on silica gel (230‐400 mesh, Merck). Yields refer to spectroscopically and analytically pure compounds unless otherwise stated. ^1^H‐NMR spectra were recorded on a Varian Gemini 200 MHz, a Varian Mercury 400 MHz or on a Varian INOVA 400 MHz instruments at 25 °C. ^13^C‐NMR spectra were recorded at 50 MHz or at 100 MHz. Chemical shifts are reported relative to CDCl_3_ (^1^H: δ=7.27 ppm, ^13^C: δ=77.0 ppm). Chemical shifts are reported relative to CD_3_OD (^1^H: δ=4.87 ppm, ^13^C: δ=49.0 ppm). Integrals are in accordance with assignments, coupling constants are given in Hz. For detailed peak assignments 2D spectra were measured (g‐COSY, g‐HSQC) and 1D‐NOESY. The following abbreviations were used to designate multiplicities: s=singlet, d=doublet, t=triplet, q=quartet, m=multiplet, quin=quintuplet, sext=sextet, sept=septet, br s=broad singlet and dd=double‐doublet. IR spectra were recorded with a IRAffinity‐1S Shimadzu spectrophotometer. ESI‐MS spectra were recorded with a Thermo Scientific™ LCQ fleet ion trap mass spectrometer. Elemental analyses were performed with a Thermo Finnigan FLASH EA 1112 CHN/S analyzer. Optical rotation measurements were performed on a JASCO DIP‐370 polarimeter. The assignment of H and C atoms in NMR characterizations reflects the numbering of chemical structures in Scheme 5 and in the Supporting Information for practical reasons.

**Synthesis of (3*R*,5*R*)‐1‐(9‐azidononyl)‐3,4,5‐trihydroxypiperidine (5)**: To a solution of **4** (152 mg, 0.45 mmol) in 18 mL of MeOH, 200 μL of 37 % HCl were added and the mixture was stirred at room temperature for 18 hours. After a TLC analysis (CH_2_Cl_2_ : MeOH 20 : 1) showed disappearance of the starting material (*R*_f_=0.44), the solvent was removed under reduced pressure. The crude was purified by FCC (CH_2_Cl_2_:MeOH:NH_3_ from 10 : 1:0.1 to 6 : 1:0.1) affording pure **4** (*R*_f_=0.19, 132 mg, 0.44 mmol, 99 % yield) as a colorless oil. [α] _D_^21^=−21.6 (c=1.02 in MeOH); ^1^H‐NMR (400 MHz, CD_3_OD): δ= 3.91 (q, *J*=5.8 Hz, 1H, H‐3), 3.80 (td, *J*=7.9, 4.0 Hz, 1H, H‐5), 3.42–3.39 (m, 1H, H‐4), 3.29 (t, *J*=6.8 Hz, 2H, H‐1’), 2.84–2.76 (m, 2H, Ha‐6, Ha‐2), 2.44–2.32 (m, 2H, Hb‐6, Hb‐2), 2.29 (d, *J*=12.1 Hz, 1H, Ha‐9’), 2.15–2.06 (m, 1H, Hb‐9’), 1.64–1.57 (m, 2H, H‐2’), 1.54–1.51 (m, 2H, H‐8’), 1.35 (bs, 10H, from H‐3’ to H‐7’) ppm; ^13^C‐NMR (50 MHz, CD_3_OD): δ=75.3 (C‐4), 69.6 (C‐5), 69.1 (C‐3), 59.3 (C‐9’), 58.2 (C‐2), 57.6 (C‐6), 52.4 (C‐1’), 30.5–27.5 (7 C, from C‐2’ to C‐8’) ppm; IR (CD_3_OD): ν=3690, 3585, 3429, 2932, 2856, 2817, 2098, 1469, 1068 cm‐1. MS (ESI): m/z calcd (%) for C_14_H_28_N_4_O_3_: 300.22; found: 301.33 (100 %, [M+H]^+^). Elemental analysis: C_14_H_28_N_4_O_3_ (300.40) calcd. C, 55.98; H, 9.40; N, 18.65; found C, 56.02; H, 9.32, N, 18.55.

**General procedure for CuAAC reaction to synthesize multivalent compounds**: To a solution of **5** (appropriate equivalent) in a 2 : 1 THF:H_2_O mixture and alkyne **6–10** (1 equiv.), CuSO_4_ (0.3 equiv) and sodium ascorbate (0.6 equiv) were added. The reaction mixture was stirred in a MW reactor at 80 °C for 45 min until TLC analysis (DCM: MeOH 10 : 1) showed the disappearance of the starting material (*R*_f_=0.21) and formation of the desired product. After filtration through Celite®, the solvent was removed under reduced pressure and the crude was first treated with Quadrasil® MP resin then purified through SEC (with Sephadex LH‐20), using water as a solvent, to obtain the multivalent adducts **11–15**.

**Aromatic trivalent 12**: The general procedure employing scaffold **7** (1 equiv.) and 3.3 equiv. of **5** afforded 89 % yield of **12** as a white waxy solid (48 mg, 0.04 mmol). [α] _D_^26^=−17.5 (c=1.02 in CH_3_OH); ^1^H‐NMR (400 MHz, CD_3_OD): δ=8.46 (s, 3H, H−Ar), 8.29 (s, 3H, H triazole), 4.49 (t, *J*=7.0 Hz, 6H, H‐9’), 3.89 (dd, *J*=5.5, 2.8 Hz, 3H, H‐3), 3.80 (td, *J*=7.7, 3.9 Hz, 3H, H‐5), 3.45‐3.38 (m, 3H, H‐4), 2.83‐2.76 (m, 6H, Ha‐6, Ha‐2), 2.41‐2.33 (m, 6H, H‐1’), 2.31‐2.28 (m, 3H, Hb‐2), 2.18‐2.05 (m, 3H, Hb‐6), 2.02‐1.96 (m, 6H, H‐8’), 1.54‐1.44 (m, 6H, H‐2’), 1.36‐1.30 (m, 30H, from H‐3’ to H‐7’) ppm; ^13^C‐NMR (50 MHz, CD_3_OD): δ= 148.0 (3 C, *C*‐Ar triazole), 133.4 (3 C, *C*‐Ar), 123.3 (3 C, *C*H−Ar), 122.8 (3 C, *C*H triazole), 75.0 (3 C, C‐4), 69.4 (3 C, C‐5), 68.8 (3 C, C‐3), 59.2 (3 C, C‐1’), 57.9 (3 C, C‐2), 57.3 (3 C, C‐6), 51.6 (3 C, C‐9’), 31.2 (3 C, C‐8’) 30.4‐27.5 (15 C, from C‐3’ to C‐7’) 27.3 (3 C, C‐2’) ppm; MS (ESI): *m/z* calcd (%) for C_54_H_90_N_12_O_9_ 1050.70; found: 1073.92 (73, [M+Na]^+^), 526.58 (100, [(M/2)+H]^+^), 351.42 (59, [(M/3)+H]^+^). Elemental analysis: C_54_H_90_N_12_O_9_ (1051.37) calcd. C, 61.69; H, 8.63; N, 15.99; found C, 61.45; H, 8.35, N, 16.08.

### Biological studies

**Inhibitory activity towards human GCase**: the compounds **11–15** and **18** were screened towards GCase from leukocytes isolated from healthy donors (controls). Isolated leukocytes were disrupted by sonication, and a micro BCA protein assay kit (Sigma‐Aldrich) was used to determine the total protein amount for the enzymatic assay, according to the manufacturer instructions. Enzyme activity was measured in a flat‐bottomed 96‐well plate. Compound solution (3 μL), 4.29 μg/μL leukocytes homogenate (7 μL), and substrate 4‐methylumbelliferyl‐β‐D‐glucoside (3.33 mM, 20 μL, Sigma‐Aldrich) in citrate/phosphate buffer (0.1:0.2, M/M, pH 5.8) containing sodium taurocholate (0.3 %) and Triton X‐100 (0.15 %) at 37 °C were incubated for 1 h. The reaction was stopped by addition of sodium carbonate (200 μL; 0.5 M, pH 10.7) containing Triton X‐100 (0.0025 %), and the fluorescence of 4‐methylumbelliferone released by β‐glucosidase activity was measured in SpectraMax M2 microplate reader (λ_ex_=365 nm, λ_em_=435 nm; Molecular Devices). Percentage GCase inhibition is given with respect to the control (without iminosugar). Data are mean ± SD (n=3).

**IC_50_ determination**: The IC_50_ values of inhibitors **11–15** and **18** against GCase from leukocytes isolated from healthy donors were determined by measuring the initial hydrolysis rate with 4‐methylumbelliferyl‐β‐d‐glucoside (3.33 mM). Data are mean ± SD (n=3). Data obtained were fitted using the Origin Microcal program (see the Supporting Information file for further details). The IC_50_ value of **12** against recombinant wild‐type human GCase enzyme (VPRIV®) was also determined.

**Kinetic analysis for compound 12**: The action mechanism of compound **12** was determined studying the dependence of the main kinetic parameters (K_M_ and V_max_) from the inhibitor concentration. Kinetic data were analyzed using the Lineweaver‐Burk plot (for more details, see Supporting Information file).

**Chaperoning activity assays**: The effect of multimeric iminosugars (**12**, **13** and **15**) on GCase activity was evaluated in Gaucher patients’ cells fibroblasts with the N370S/RecNcil (or L444P/L444P) mutations. Gaucher disease patients’ cells were obtained from the “Cell line and DNA Biobank from patients affected by Genetic Diseases” (Gaslini Hospital, Genova, Italy). Fibroblasts cells (20×10^4^) were seeded in T25 flasks with DMEM supplemented with fetal bovine serum (10 %), penicillin/streptomycin (1 %), and glutamine (1 %) and incubated at 37 °C with 5 % CO_2_ for 24 h. The medium was removed, and fresh medium containing the multimeric iminosugars was added to the cells and left for 4 days. The medium was removed, and the cells were washed with PBS and detached with trypsin to obtain cell pellets, which were washed four times with PBS, frozen and lysed by sonication in water. Enzyme activity was measured as reported above. Reported data are mean ± S.D. (n=2).

All experiments on biological materials were performed in accordance with the ethical standards of the institutional research committee and with the 1964 Helsinki Declaration and its later amendments. Control and patient samples were anonymized and used only for research purposes for which written informed consent had been obtained using a form approved by the local Ethics Committee.

**Thermal stabilization assay**:^[32]^ Recombinant wild‐type human GCase enzyme (VPRIV® 1.0×10^−9^ mg/mL) aliquots (100 μL) with 0 (control), 1, 10, 100, 200 μM of compound **12** were incubated at pH 7.0 for 20 minutes at 0 °C and then heated at 48 °C for 0 minutes, 20 minutes, 40 minutes or 60 minutes. Subsequently, 100 μL of water were added in each aliquot. Then each 10 μL aliquot was incubated with 20 μL of substrate 4‐methylumbelliferyl‐β‐d‐glucoside (3.33 mM, Sigma‐Aldrich) in citrate/phosphate buffer (0.1:0.2, M/M, pH 5.8) containing sodium taurocholate (0.3 %) and Triton X‐100 (0.15 %) at 37 °C, for 1 h. The reaction was stopped by addition of sodium carbonate (200 μL; 0.5 M, pH 10.7) containing Triton X‐100 (0.0025 %), and the fluorescence of 4‐methylumbelliferone released by GCase activity was measured in SpectraMax M2 microplate reader (λ_ex_=365 nm, λ_em_=435 nm; Molecular Devices). Data are mean SD ± (n=3). The graph of GCase activity after different heating times was reported in the Supporting Information file. The graph of GCase activity calculated with respect to the not heated control (Relative GCase activity) was reported in Figure 4.

## Conflict of interest

The authors declare no conflict of interest.

1

## Supporting information

As a service to our authors and readers, this journal provides supporting information supplied by the authors. Such materials are peer reviewed and may be re‐organized for online delivery, but are not copy‐edited or typeset. Technical support issues arising from supporting information (other than missing files) should be addressed to the authors.

Supporting Information

## Data Availability

The data that support the findings of this study are available in the supplementary material of this article.
