# Supplementary material for: 3,4,5‐Trihydroxypiperidine Based Multivalent Glucocerebrosidase (GCase) Enhancers
Source: Chembiochem. 2022 Apr 7;23(11):e202200077. doi: 10.1002/cbic.202200077 (PMC9400994; doi:10.1002/cbic.202200077)
Supplement: Supplementary file 1 — Supporting Information [file CBIC-23-0-s001.pdf]

ChemBioChem

Supporting Information

3,4,5-Trihydroxypiperidine Based Multivalent Glucocerebrosidase (GCase) Enhancers

Costanza Vanni[+], Francesca Clemente[+], Paolo Paoli, Amelia Morrone, Camilla Matassini,* Andrea Goti, and Francesca Cardona*

Table of contents

Synthesis, ^{1}H-NMR and ^{13}C-NMR spectra of compound **4** (Figures S1-S2) S3

^{1}H-NMR and ^{13}C-NMR spectra of compound **5** (Figures S3-S4) S5

Synthesis, ^{1}H-NMR and ^{13}C-NMR spectra of compound **11** (Figures S5-S6) S6

^{1}H-NMR and ^{13}C-NMR spectra of compound **12** (Figures S7-S8) S8

Synthesis, ^{1}H-NMR and ^{13}C-NMR spectra of compound **13** (Figures S9-S10) S10

Synthesis, ^{1}H-NMR and ^{13}C-NMR spectra of compound **14** (Figures S11-S12) S12

Synthesis, ^{1}H-NMR and ^{13}C-NMR spectra of compound **15** (Figures S13-S14) S14

Synthesis, ^{1}H-NMR and ^{13}C-NMR spectra of compound **17** (Figures S15-S16) S16

Synthesis, ^{1}H-NMR and ^{13}C-NMR spectra of compound **18** (Figures S17-S18) S18

Enzymatic assay (Figure S19) S20

IC_{50} graphs of compounds **11** and **12** (Figures S20-S21) S21

IC_{50} graphs of compounds **13** and **14** (Figures S22-S23) S22

IC_{50} graphs of compounds **15** and **18** (Figures S24-S25) S23

IC_{50} determination of **12** towards recombinant wild-type human GCase S24

K_i determination of **12** S25

Chaperoning activity assays S27

Thermal Stabilization Assay S29

Synthesis of (3*R*,4*S*,5*R*)-1-(9-Azidononyl)-5-hydroxy-3,4-(isopropylidenedioxy)-piperidine (4): A solution of **2** (63 mg, 0.36 mmol), 1-azido-6-bromohexane (**3**, 135 mg, 0.55 mmol) and K_2CO_3 (75 mg, 0.55 mmol) in 2.4 ml of a 5:1 CH_3CN/H_2O mixture was stirred in microwave at 120°C for 2 h, until a TLC analysis (CH_2Cl_2:MeOH:NH_3 6:1:0.1) showed the disappearance of the starting material and the formation of a new product (*R*f = 0.45). After filtration through Celite®, the solvent was removed under reduced pressure and the crude was purified by FCC (EtOAc: PE from 1:1 to 2:1) affording pure **4** (*R*f = 0.18, 89 mg, 0.26 mmol, 73% yield) as a yellow oil. $[\alpha]_D^{21}$= −30.7 (c = 0.79 in MeOH); ^{1}H-NMR (400 MHz, CD_3OD): δ= 4.30 (dd, *J* = 8.3, 3.8 Hz, 1H, H-5), 3.89-3.77 (m, 2H, H-3, H-4), 3.29 (t, *J* = 6.8 Hz, 2H, H-1'), 3.04 (d, *J* = 13.1 Hz, 1H, Ha-6), 2.76 (dd, *J* = 12.0, 4.0 Hz, 1H, Ha-2), 2.44 (dd, *J* = 13.3, 3.9 Hz, 1H, Hb-6), 2.40-2.38 (m, 2H, H-9'), 2.02 (dd, *J* = 11.4, 9.0 Hz, 1H, Hb-2), 1.62-1.36 m, 14H, from H-2' to H-8'), 1.51 (s, 3H, CH_3), 1.36 (s, 3H, CH_3) ppm; ^{13}C-NMR (50 MHz, CD_3OD): δ= 110.1 (O*C*(CH_3)$_2$), 80.2 (C-4), 74.5 (C-3), 70.4 (C-5), 59.2 (C-9'), 57.7 (C-6), 55.1 (C-2), 52.4 (C-1'), 30.5-27.8 (7C from C-2' to C-8'), 27.5-26.6 (2C, OC(*C*H_3)$_2$) ppm; IR (CD_3OD): ν = 3022, 2989, 2934, 2856, 2098, 1470, 1383, 1225, 1059 cm^{-1}. MS (ESI): *m/z* calcd (%) for $C_{17}H_{32}N_4O_3$: 340.25; found: 341.25 (100%, $[M+H]^+$), 363.25 (71%, $[M+Na]^+$). Elemental analysis calcd for $C_{17}H_{32}N_4O_3$: C 59.97, H 9.47, N 16.46, found: C 59.87, H 9.50, N 16.52.

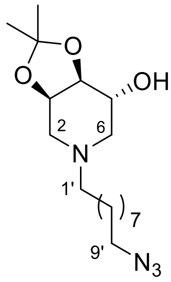

4

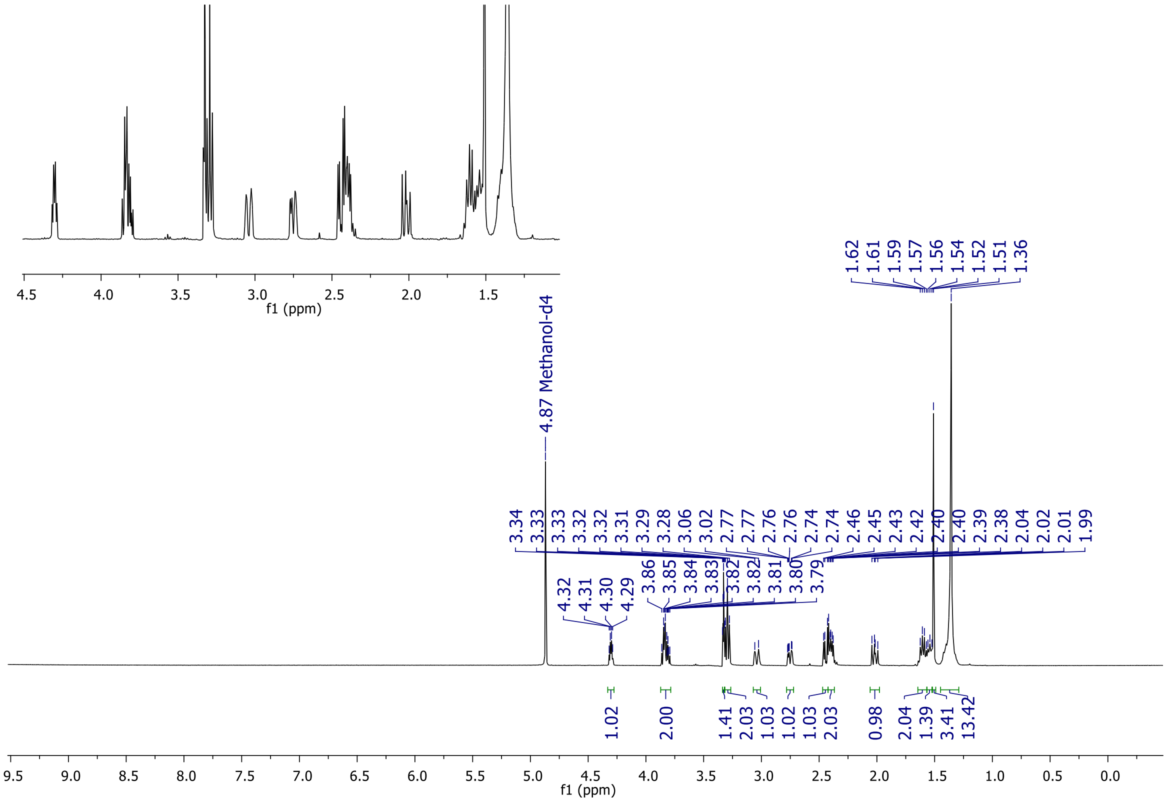

Figure S1: ^{1}H-NMR spectrum of compound **4** (400 MHz, CD_3OD).

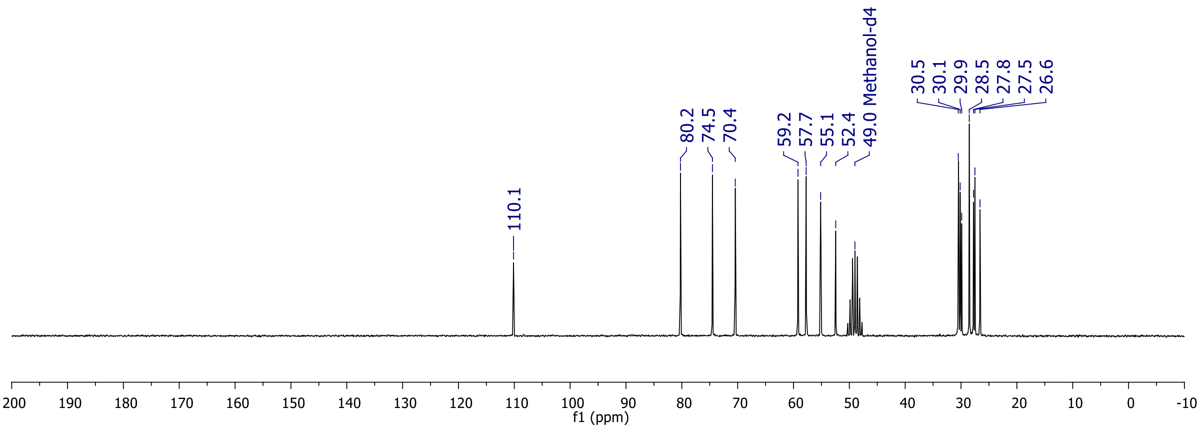

Figure S2: ^{13}C-NMR spectrum of compound **4** (50 MHz, CD_3OD).

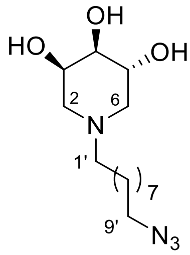

5

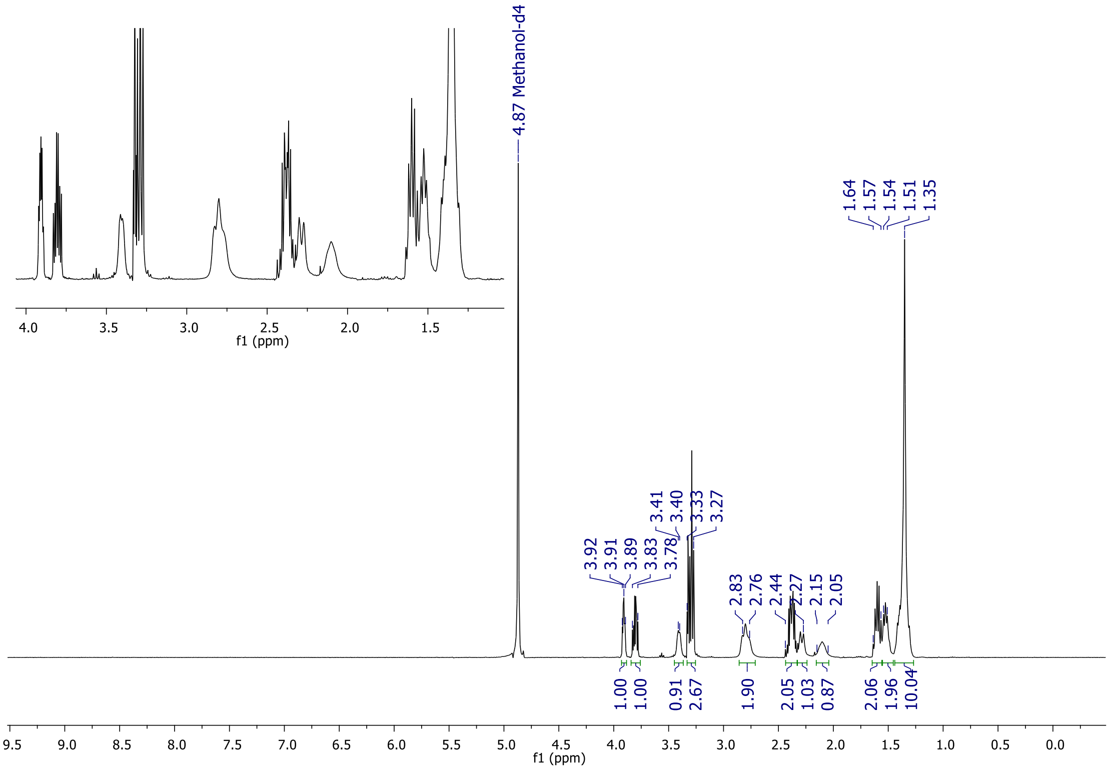

Figure S3: ^{1}H-NMR spectrum of compound **5** (400 MHz, CD_3OD).

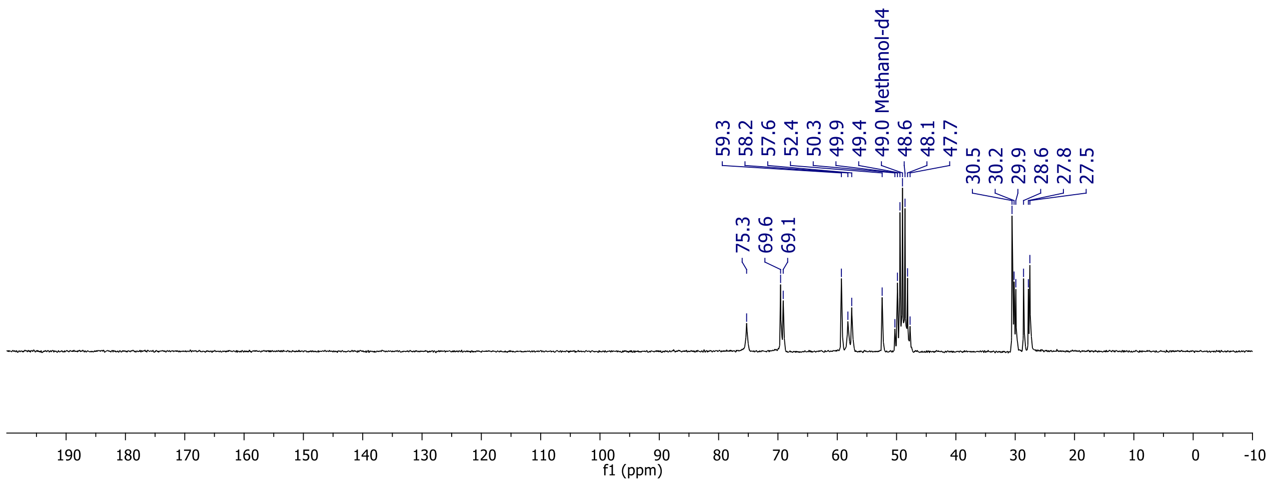

Figure S4: ^{13}C-NMR spectrum of compound **5** (50 MHz, CD_3OD).

Compound 11: Application of the general procedure for CuAAC reaction (reported in the main text) to scaffold **6** (1 equiv.) and 3.3 equiv. of **5** afforded 55% yield of **11** as a white waxy solid (47 mg, 0.04 mmol). $[\alpha]_D^{26}$= -12.3 (c = 1.03 in CH_3OH); ^{1}H-NMR (400 MHz, CD_3OD): δ= 7.98 (s, 3H, H triazole), 4.59 (bs, 6H, C*H2*-O), 4.40 (t, *J* = 7.0 Hz, 6H, H-9'), 3.99-3.95 (m, 3H, H-3), 3.87-3.83 (m, 3H, H-5), 3.55-3.45 (m, 9H, H-4, C*H2*-O), 3.35 (s, 2H, NH_2), 2.93-2.86 (m, 6H, Ha-6, Ha-2), 2.55-2.47 (m, 9H, H-1', Hb-2), 2.38-2.28 (m, 3H, Hb-6), 1.94-1.89 (m, 6H, H-8'), 1.56-1.55 (m, 6H, H-2'), 1.33 (bs, 30H, from H-3' to H-7') ppm; ^{13}C-NMR (50 MHz, CD_3OD): δ= 145.5 (3C, *C*-Ar triazole), 125.0 (3C, *C*H triazole), 74.3 (3C, *C*H_2-O), 71.5 (3C, C-4), 69.1 (3C, C-5), 68.3 (3C, C-3), 65.4 (3C, *C*H_2-O), 59.0 (3C, C-1'), 57.3 (H_2N*C*$(CH_2O)_3$), 56.8 (6C, C-2, C-6), 51.4 (3C, C-9'), 29.9 (3C, C-8'), 31.2-27.4 (15C, from C-3' to C-7'), 26.9 (3C, C-2') ppm; MS (ESI): *m/z* calcd (%) for $C_{55}H_{101}N_{13}O_{12}$ 1136.47; found: 1159.00 (100, $[M+Na]^+$), 379.83 (77, $[(M/3)+H]^+$). Elemental analysis: $C_{55}H_{101}N_{13}O_{12}$ (1136.47) calcd. C, 58.13; H, 8.96; N, 16.02; found C, 58.19; H, 9.01, N, 15.89.

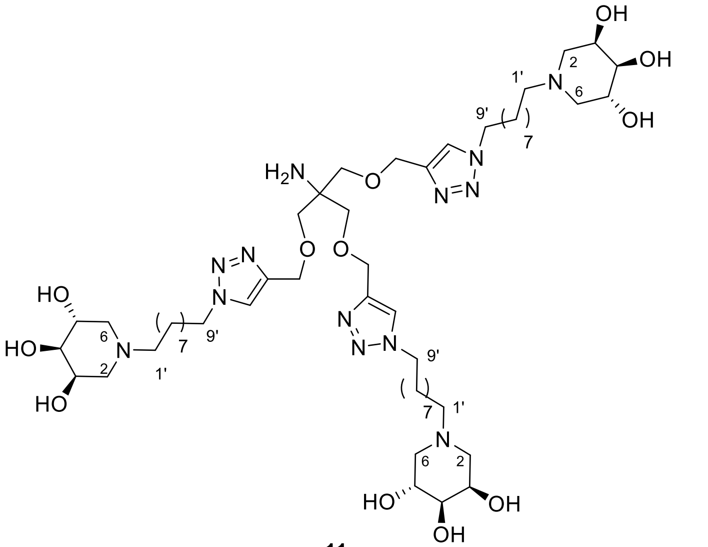

11

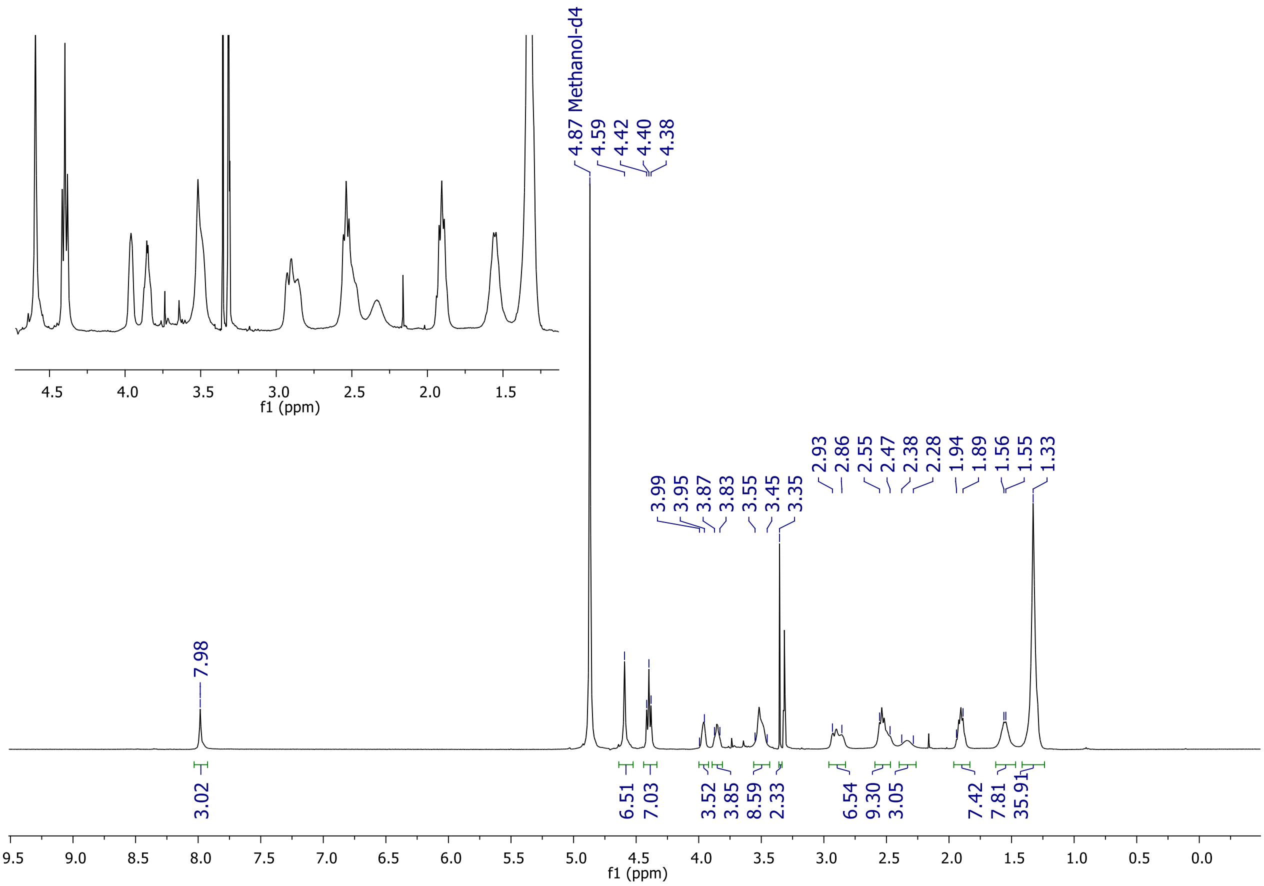

Figure S5: ^{1}H-NMR spectrum of compound **11** (400 MHz, CD_3OD).

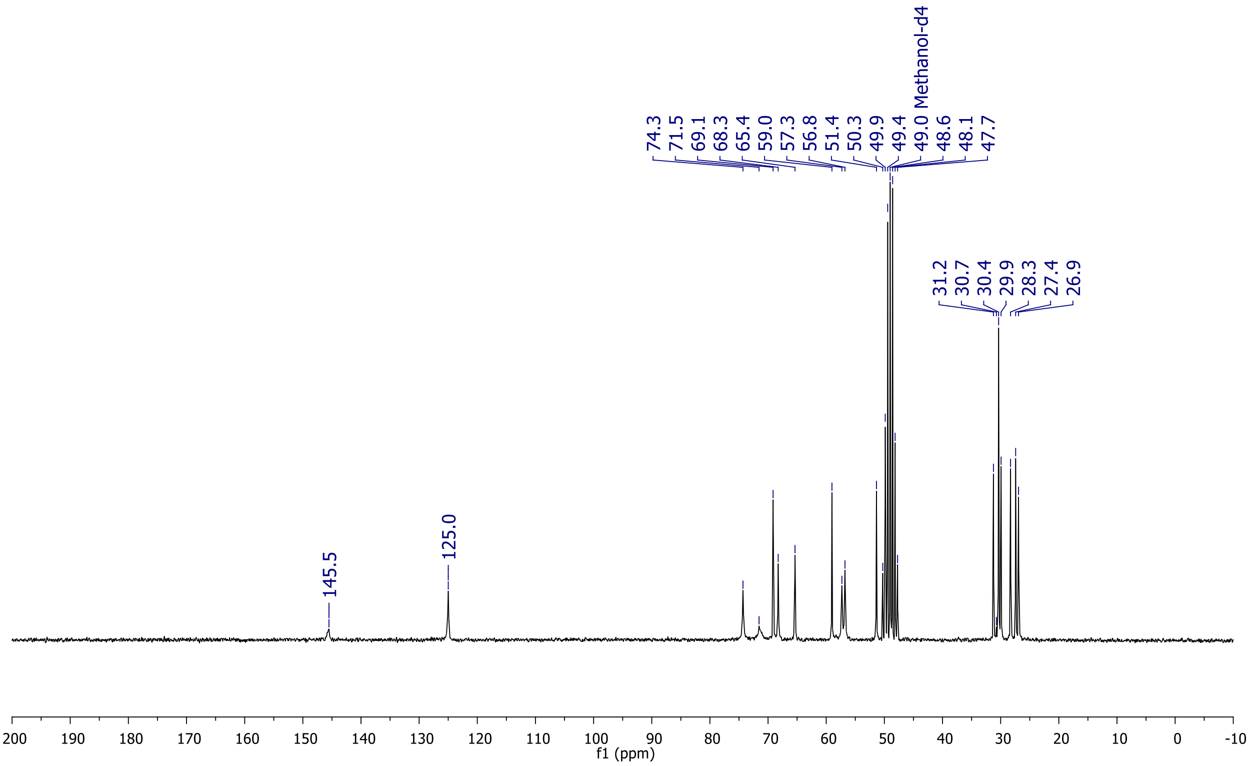

Figure S6: ^{13}C-NMR spectrum of compound **11** (50 MHz, CD_3OD).

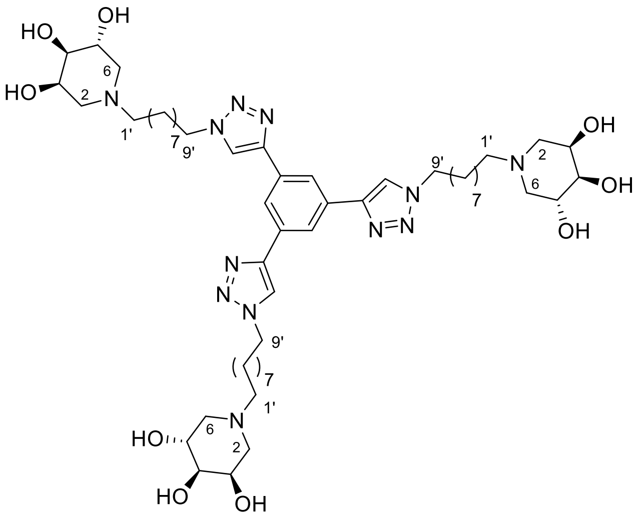

12

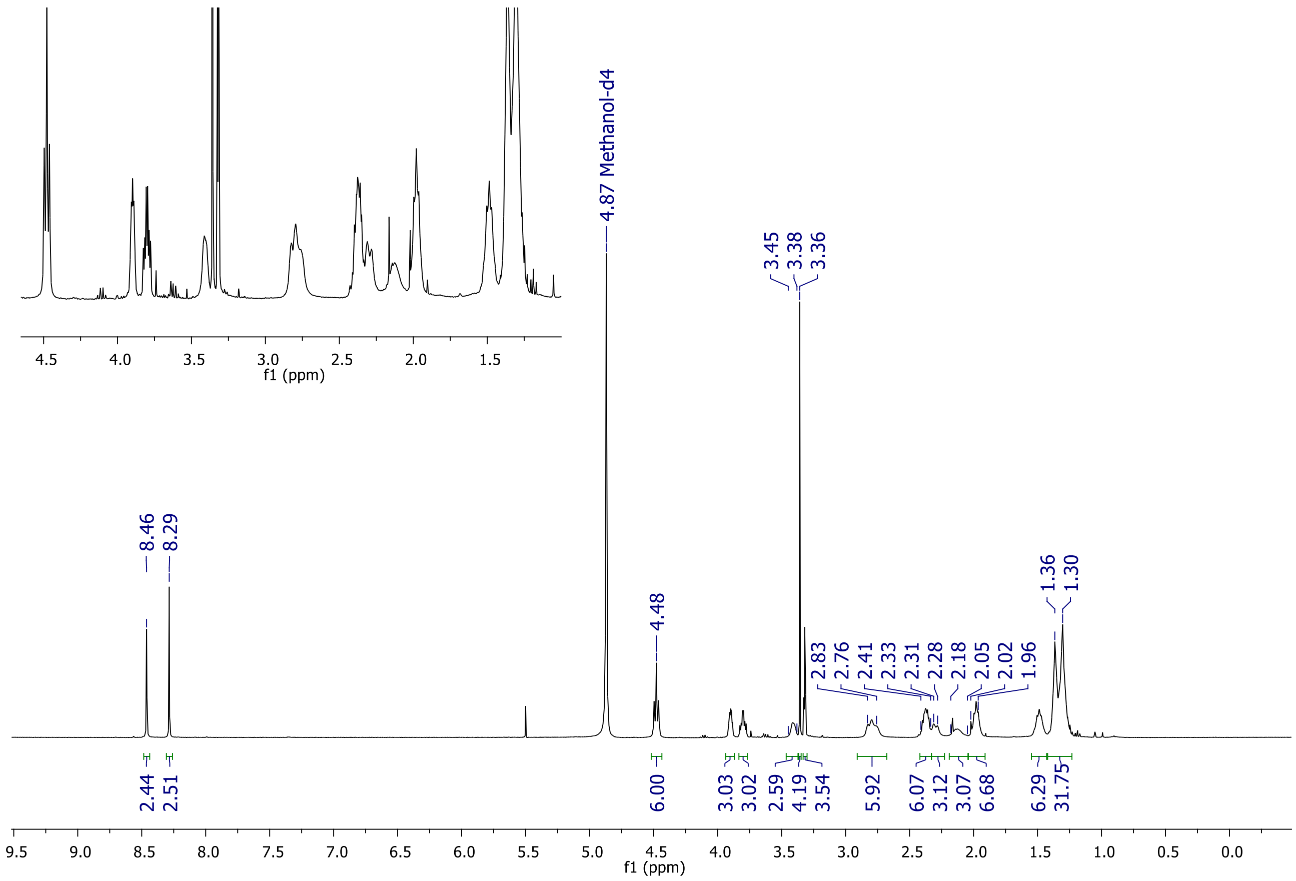

Figure S7: ^{1}H-NMR spectrum of compound **12** (400 MHz, CD_3OD).

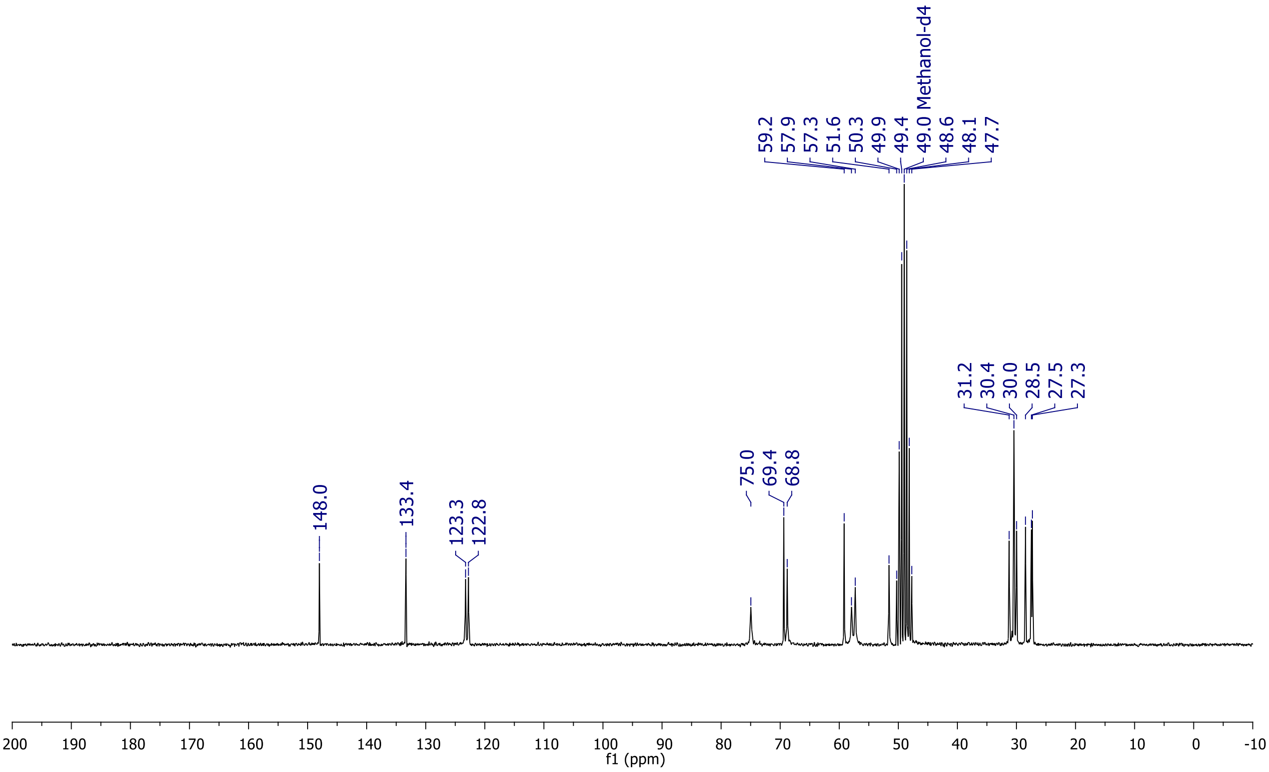

Figure S8: ^{13}C-NMR spectrum of compound **12** (50 MHz, CD_3OD)

Tetravalent 13: Application of the general procedure for CuAAC reaction (reported in the main text) to scaffold **8** (1 equiv.) and 4.4 equiv. of **5** afforded 61% of **13** as a white waxy solid (45 mg, 0.03 mmol). $[\alpha]_D^{23}$= -14.9 (c = 0.98 in CH_3OH); ^{1}H-NMR (400 MHz, CD_3OD): δ= 7.98 (s, 4H, H triazole), 4.51 (s, 8H, C*H2*-O), 4.39 (t, *J* = 7.1 Hz, 8H, H-9'), 3.95 (dd, *J* = 3.9, 1.3 Hz, 4H, H-3), 3.84 (td, *J* = 7.1, 3.7 Hz, 4H, H-5), 3.47-3.44 (m, 12H, H-4, C*H2*-O), 2.90-2.83 (m, 8H, Ha-6, Ha-2), 2.51-2.42 (m, 12H, H-1', Hb-2), 2.32-2.23 (m, 4H, Hb-6), 1.94-1.87 (m, 8H, H-8'), 1.60-1.47 (m, 8H, H-2'), 1.32 (bs, 40H, from H-3' to H-7') ppm; ^{13}C-NMR (50 MHz, CD_3OD): δ= 146.2 (4C, *C*-Ar triazole), 124.8 (4C, *C*H triazole), 74.6 (4C, *C*H_2-O), 70.0 (4C, C-4), 69.3 (4C, C-5), 68.5 (4C, C-3), 65.4 (4C, *C*H_2-O), 59.1 (4C, C-1'), 57.6 (4C, C-2), 57.0 (4C, C-6), 51.3 (4C, C-9'), 46.5 (*C*(CH_2O)$_4$), 31.3-27.4 (24C, from C-3' to C-8'), 27.1 (4C, C-2') ppm; MS (ESI): *m/z* calcd (%) for $C_{73}H_{132}N_{16}O_{16}$ 1489.00; found: 756.92 (100, [(M+ Na)/2]$^+$). Elemental analysis: $C_{73}H_{132}N_{16}O_{16}$ (1489.93) calcd. C, 58.85; H, 8.93; N, 15.04; found C, 58.98; H, 8.78, N, 14.49.

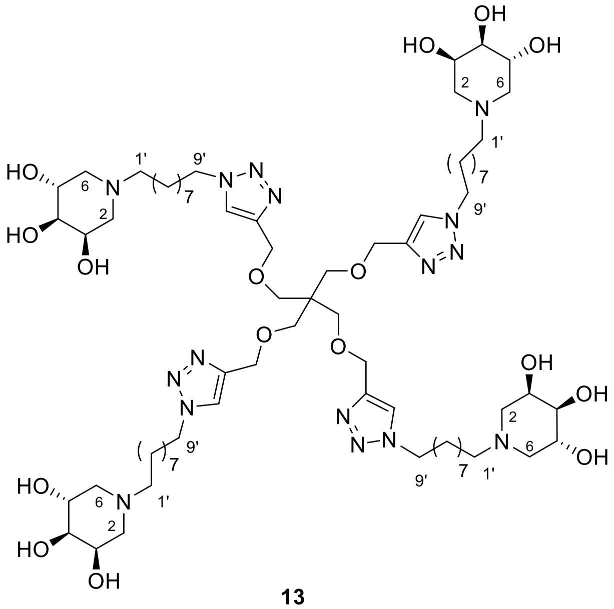

13

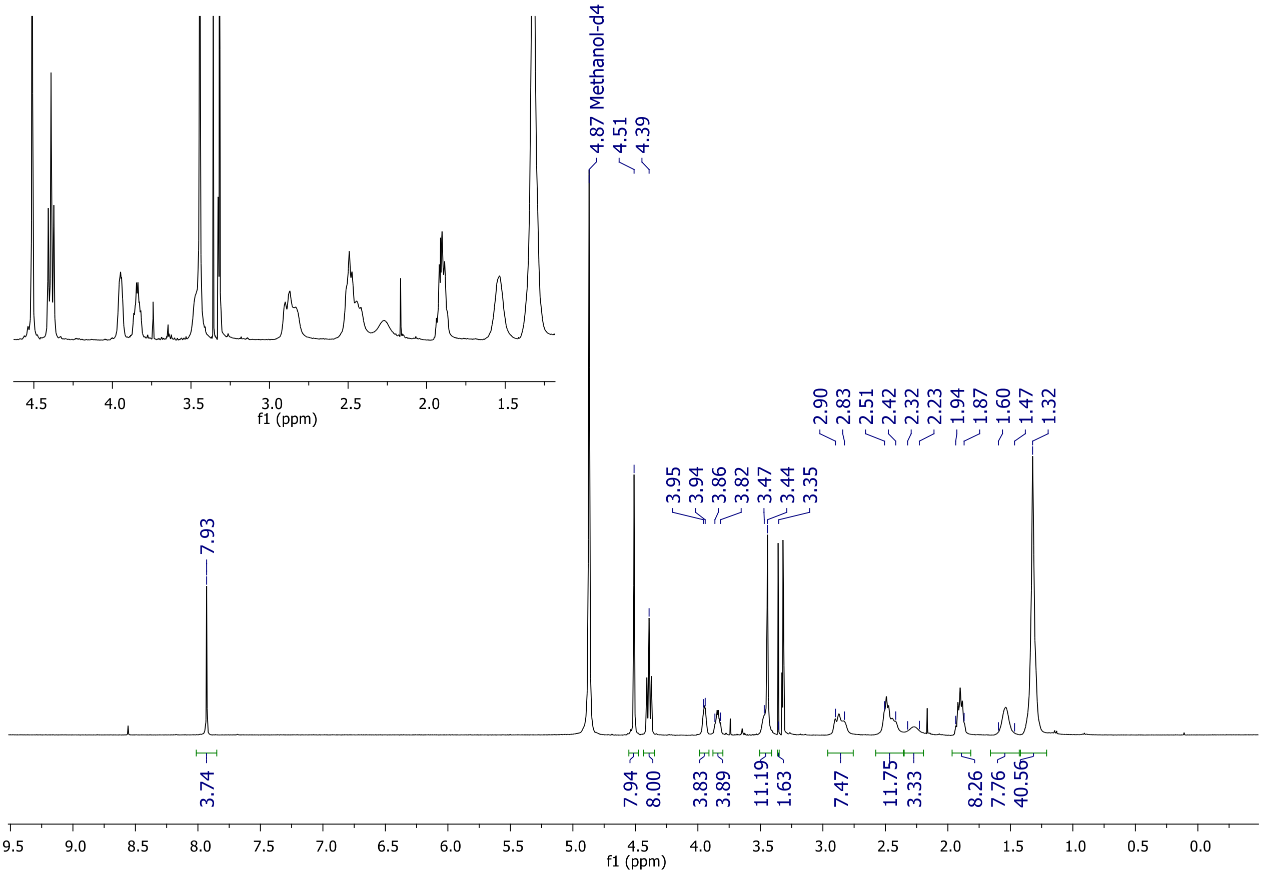

Figure S9: ^{1}H-NMR spectrum of compound **13** (400 MHz, CD_3OD).

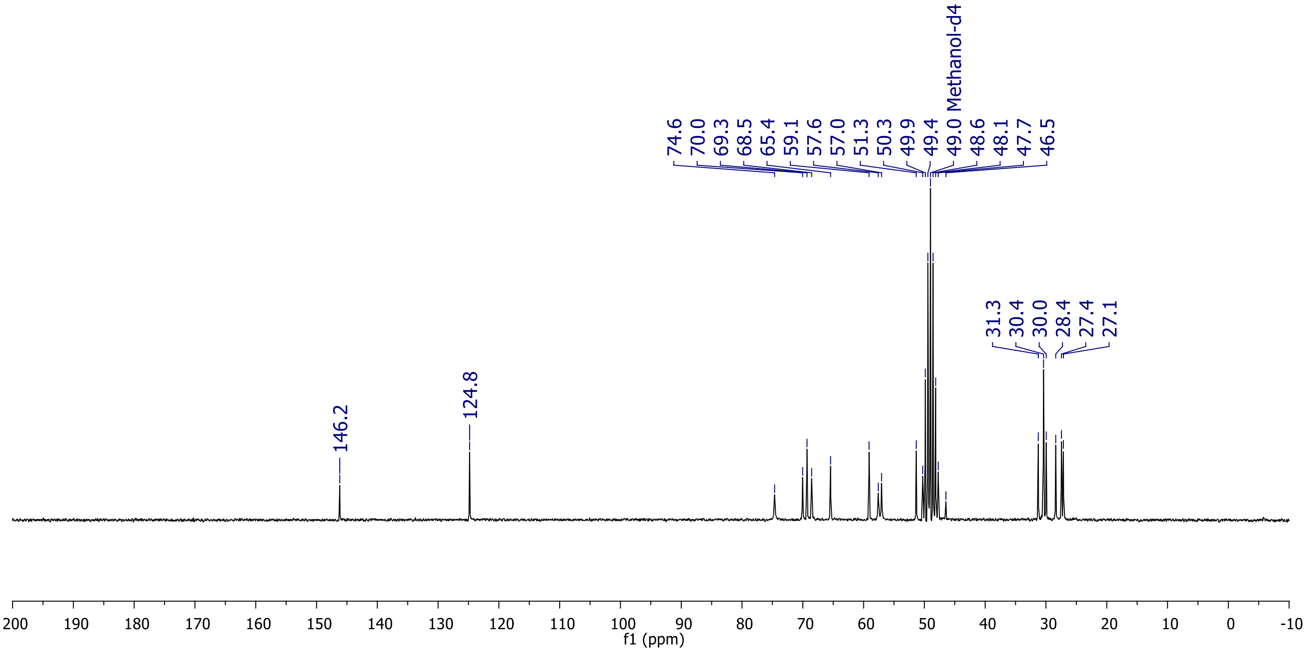

Figure S10: ^{13}C-NMR spectrum of compound **13** (50 MHz, CD_3OD).

Hexavalent 14: Application of the general procedure for CuAAC reaction (reported in the main text) to scaffold **9** (1 equiv.) and 6.6 equiv. of **5** afforded 55% of **14** as a white waxy solid (64 mg, 0.03 mmol). $[\alpha]_D^{23}$= - 7.3 (c = 0.95 in CH_3OH); ^{1}H-NMR (400 MHz, CD_3OD): δ= 8.07, 8.01, 7.97 (s, 6H, H triazole), 4.76-4.57 (s, 12H, C*H2*-O), 4.39 (dd, *J* = 13.0, 6.6 Hz, 12H, H-9'), 4.17-4.05 (m, 6H, H-3), 4.02-3.87 (m, 10H, H-5, C*H*-O), 3.85-3.65 (m, 10H, H-4, C*H2*-O), 3.24-3.06 (m, 12H, Ha-6, Ha-2), 3.05-2.74 (m, 24H, H-1', Hb-2, Hb-6), 1.97-1.77 (m, 12H, H-8'), 1.77-1.75 (m, 12H, H-2'), 1.34-1.31 (m, 60H, from H-3' to H-7') ppm; ^{13}C-NMR (100 MHz, CD_3OD): δ= 146.1 (6C, *C*-Ar triazole), 125.4 (6C, *C*H triazole), 79.6 (2C, *C*H2-O-CO), 72.3 (6C, *C*H2-O), 69.9 (6C, C-4), 68.1 (4C, *C*H-O), 66.4 (6C, C-5), 65.2 (6C, C-3), 63.7 (6C, C-1'), 55.4 (6C, C-2), 55.1 (6C, C-6), 51.3 (6C, C-9'), 31.2 (6C, C-8'), 30.1-27.4 (30C, from C-3' to C-7'), 25.6 (6C, C-2') ppm; MS (ESI): *m/z* calcd (%) for $C_{108}H_{194}N_{24}O_{24}$ 2212.47; found: 1107.01 (57, $[(M/2)+H]^+$), 738.54 (100, $[(M/3)+H]^+$), 554 (69, $[(M/4)+H]^+$). Elemental analysis: $C_{108}H_{194}N_{24}O_{24}$ (2212.84) calcd. C, 58.62; H, 8.84; N, 15.19; found C, 58.88; H, 8.61, N, 15.25.

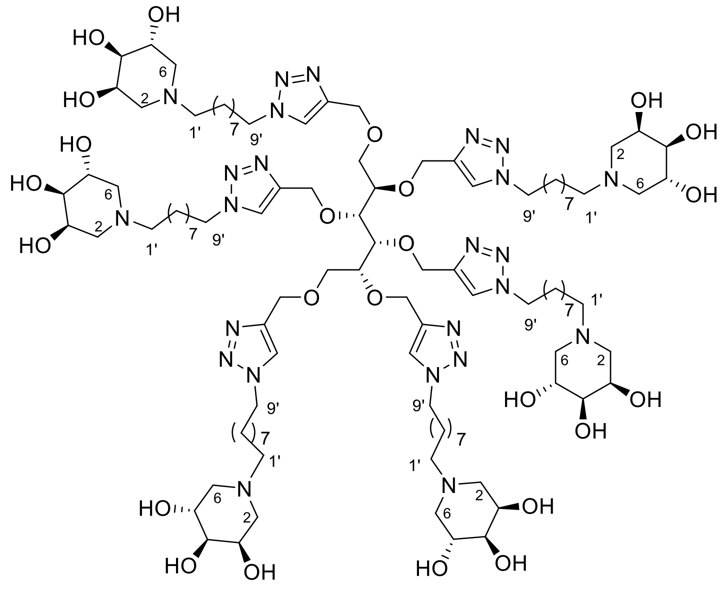

14

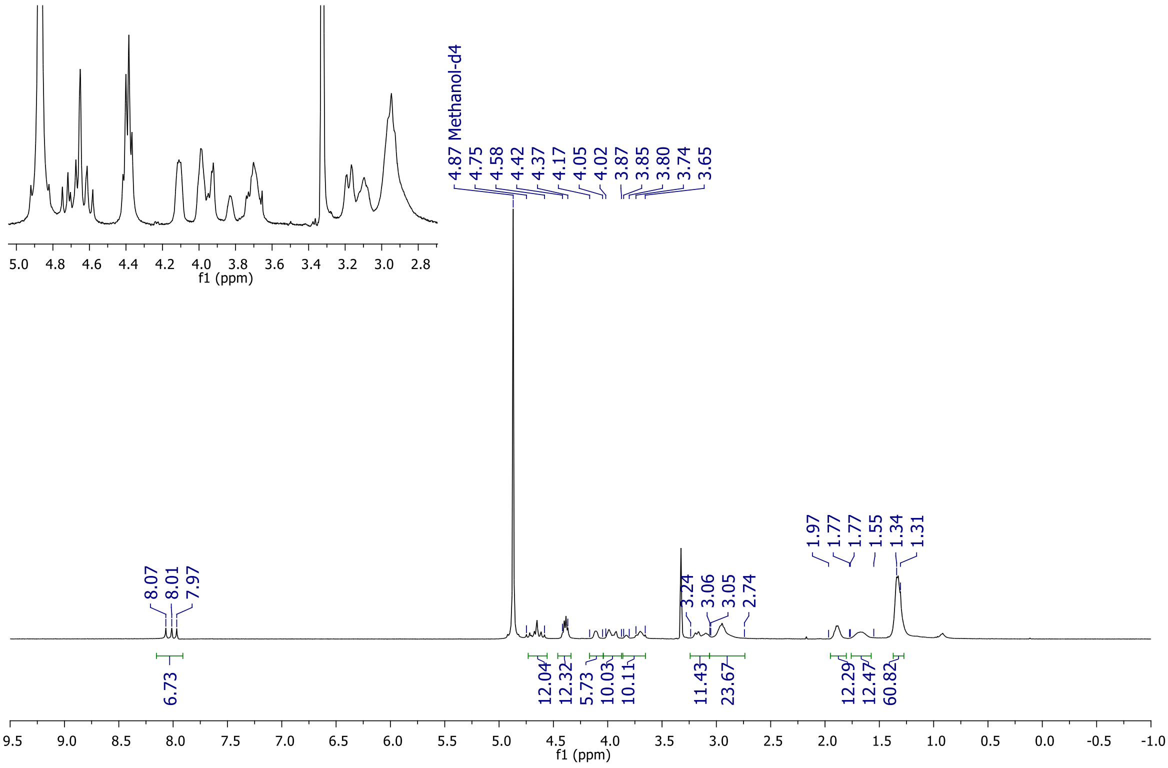

Figure S11: ^{1}H-NMR spectrum of compound **14** (400 MHz, CD_3OD).

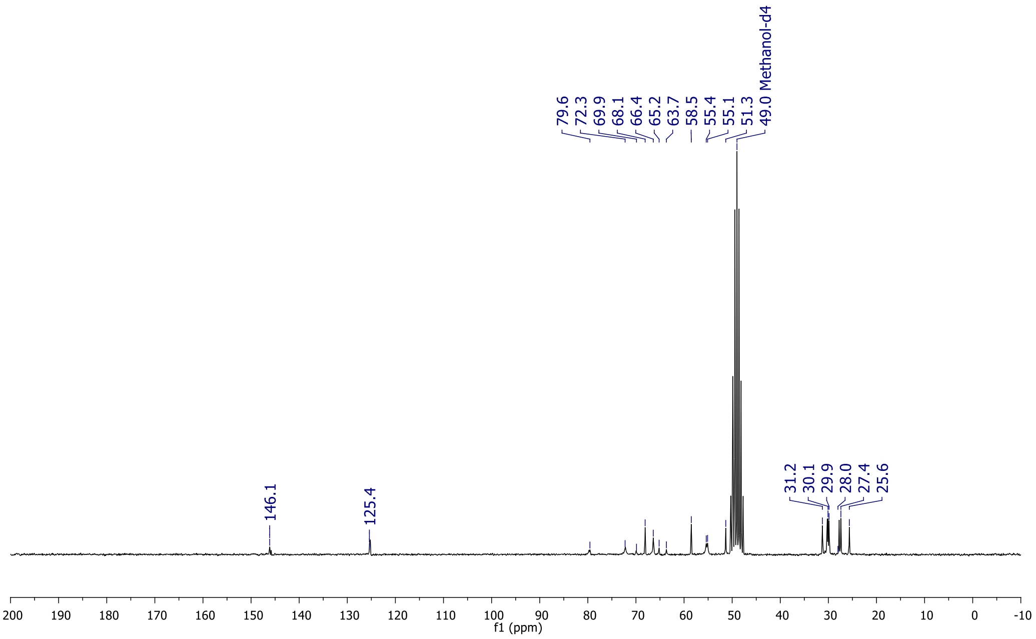

Figure S12: ^{13}C-NMR spectrum of compound **14** (100 MHz, CD_3OD).

Hexavalent 15: Application of the general procedure for CuAAC reaction (reported in the main text) to scaffold **10** (1 equiv.) and 6.6 equiv. of **5** afforded 85% of **15** as a white waxy solid (58 mg, 0.02 mmol). $[\alpha]_D^{23}$= - 16.9 (c = 0.74 in CH_3OH); ^{1}H-NMR (400 MHz, CD_3OD): δ= 7.92 (s, 6H, H triazole), 4.51 (s, 12H, C*H2*-O), 4.35 (t, *J* = 7.0 Hz, 12H, H-9'), 3.94-3.85 (m, 6H, H-3), 3.82-3.77 (m, 6H, H-5), 3.71 (s, 12H, C*H2*-O), 3.46-3.37 (m, 6H, H-4,), 2.83-2.72 (m, 12H, Ha-6, Ha-2), 2.41-2.33 (m, 18H, H-1', Hb-2), 2.25-2.11 (m, 10H, Hb-6, H-10'), 1.88-1.84 (m, 12H, H-8'), 1.57-1.41 (m, 16H, H-2', H-11'), 1.28 (bs, 60H, from H-3' to H-7') ppm; ^{13}C-NMR (100 MHz, CD_3OD): δ= 176.1 (2C, C=O), 145.8 (6C, *C*-Ar triazole), 124.9 (6C, *C*H triazole), 74.7 (6C, *C*H_2-O), 69.3 (6C, C-4), 68.6 (6C, C-5), 65.3 (6C, C-3), 61.3 (4C, C-10', C-11'), 59.2 (6C, *C*H_2-O), 57.7-57.2 (20C, C-2, C-6, C-1', CON*C*(CH_2O)$_3$), 51.3 (6C, C-9'), 31.3 (6C, C-8'), 30.5-27.3 (30C, from C-3' to C-7') 26.4 (6C, C-2') ppm; MS (ESI): *m/z* calcd (%) for $C_{116}H_{208}N_{26}O_{26}$ 2382.58; found: 1203.60 (100, $[(M+ Na)/2]^+$), (84, $[(M/2)+Na]^+$). Elemental analysis: $C_{116}H_{208}N_{26}O_{26}$ (2383.10) calcd. C, 58.46; H, 8.80; N, 15.20; found C, 58.29; H, 8.92, N, 15.46.

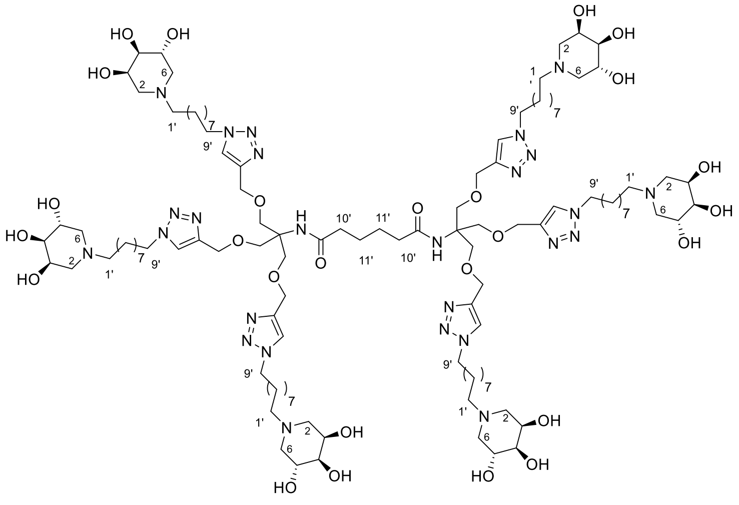

15

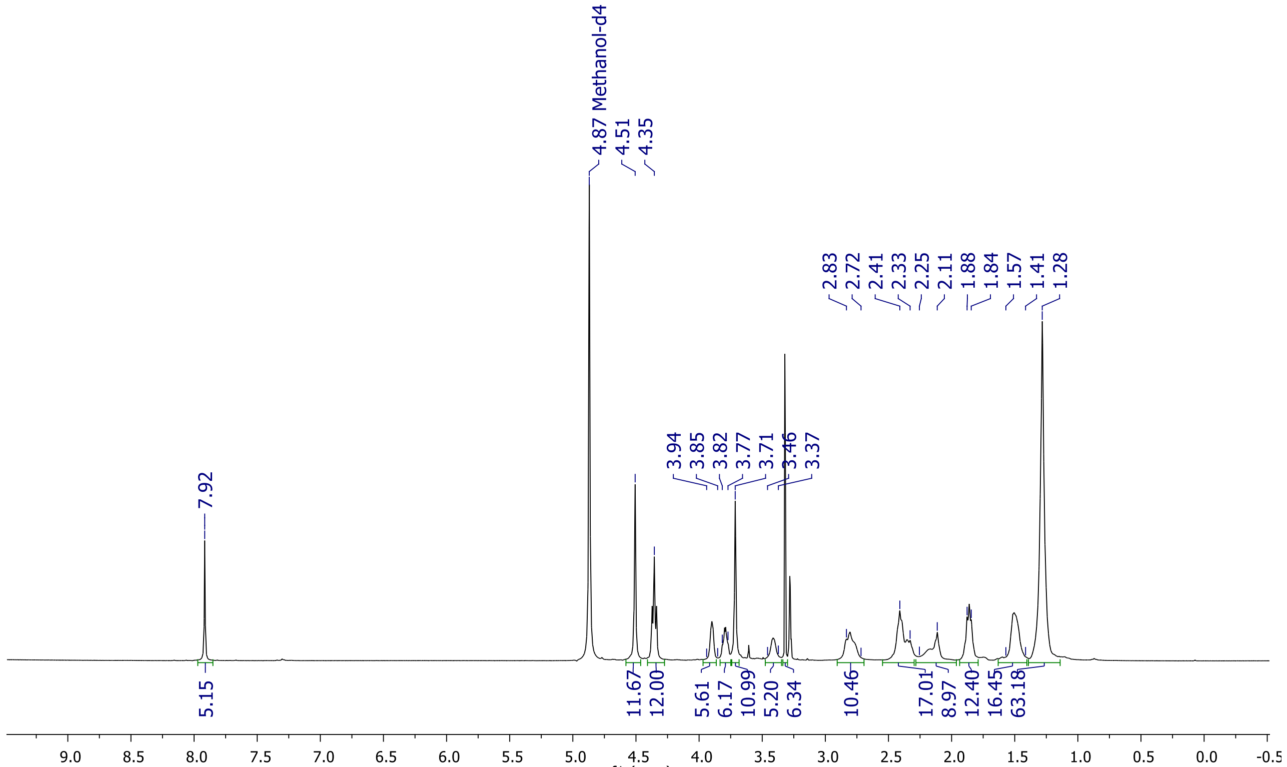

Figure S13: ^{1}H-NMR spectrum of compound **15** (400 MHz, CD_3OD).

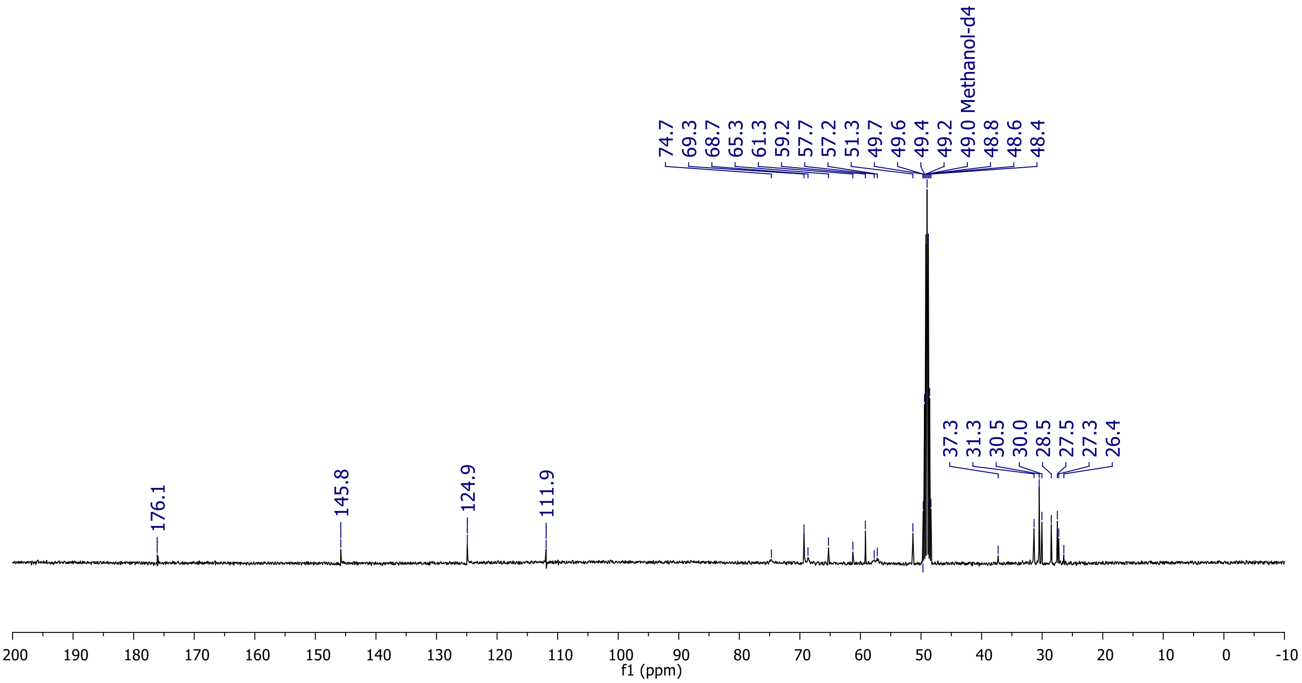

Figure S14: ^{13}C-NMR spectrum of compound **15** (100 MHz, CD_3OD).

Synthesis of compound 17: To a solution of **4** (85 mg, 0.250 mmol) in 3 ml of a 2:1 THF:H_2O mixture $CuSO_4$ (30 mol%), sodium ascorbate (60 mol%) and propargyl alcohol (**16**, 1 equiv.) were added. The reaction mixture was stirred in a MW reactor at 80 °C for 45 min until TLC analysis (DCM: MeOH 10:1) showed the disappearance of the starting material (*R*f = 0.59) and formation of a new product (*R*f = 0.00). After filtration through Celite®, the solvent was removed under reduced pressure and the crude was first treated with Quadrasil® MP resin then purified by FCC (CH_2Cl_2:MeOH:NH_3 from 30:1:0.1 to 6:1:0.1) affording pure **17** (*R*f = 0.26, 77 mg, 0.19 mmol, 78% yield) as a colourless oil. $[\alpha]_D^{25}$= + 11.5 (c = 0.90 in $CHCl_3$); ^{1}H-NMR (400 MHz, $CDCl_3$): δ= 7.51 (s, 1H, H triazole), 4.76 (s, 2H, C*H₂*OH), 4.31 (td, *J* = 7.1, 2.5 Hz, 2H, H-9'), 4.28-4.26 (m, 1H, H-5), 4.04-4.01 (m, 1H, H-4), 3.95-3.92 (m, 1H, H-3), 3.27 (bs, 1H, OH), 2.72-2.68 (m, 1H, Ha-6), 2.56 (d, *J* = 11.7 Hz, 1H, Ha-2), 2.44 (d, *J* = 11.1 Hz, 1H, Hb-2), 2.39 (dd, *J* = 12.0, 3.2 Hz, 1H, Hb-6), 2.35-2.31 (m, 2H, H-1'), 1.92-1.81 (m, 2H, H-8'), 1.48 (s, 3H, C*H₃*), 1.45-1.37 (m, 2H, H-2'), 1.34 (s, 3H, C*H₃*), 1.28-1.24 (m, 10H, from H-3' to H-7') ppm; ^{13}C-NMR (50 MHz, $CDCl_3$): δ= 147.9 (*C*-CH_2OH Ar), 121.6 (*C*H triazole), 109.5 (O*C*(CH_3)$_2$), 76.6 (C-4), 72.3 (C-5), 67.8 (C-3), 57.9 (C-1'), 56.7 (*C*H_2OH), 56.0 (C-2), 55.7 (C-6), 50.5 (C-9'), 30.4 (C-8') 29.9-27.4 (6C, from C-2' to C-7') 26.9-26.5 (2C, OC(*C*H_3)$_2$) ppm; IR ($CDCl_3$): ν = 3669, 3406, 3005, 2934, 2859, 2361, 1464, 1379, 1142, 1057, 926 cm^{-1}. MS (ESI): *m/z* calcd (%) for $C_{20}H_{36}N_4O_4$ 396.27; found: 397.53 (100%, $[M+H]^+$). Elemental analysis: $C_{20}H_{36}N_4O_4$ (396.53) calcd. C, 60.58; H, 9.15; N, 14.13; found C, 56.52; H, 9.32, N, 18.45.

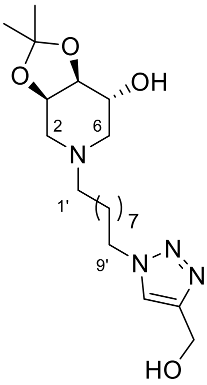

17

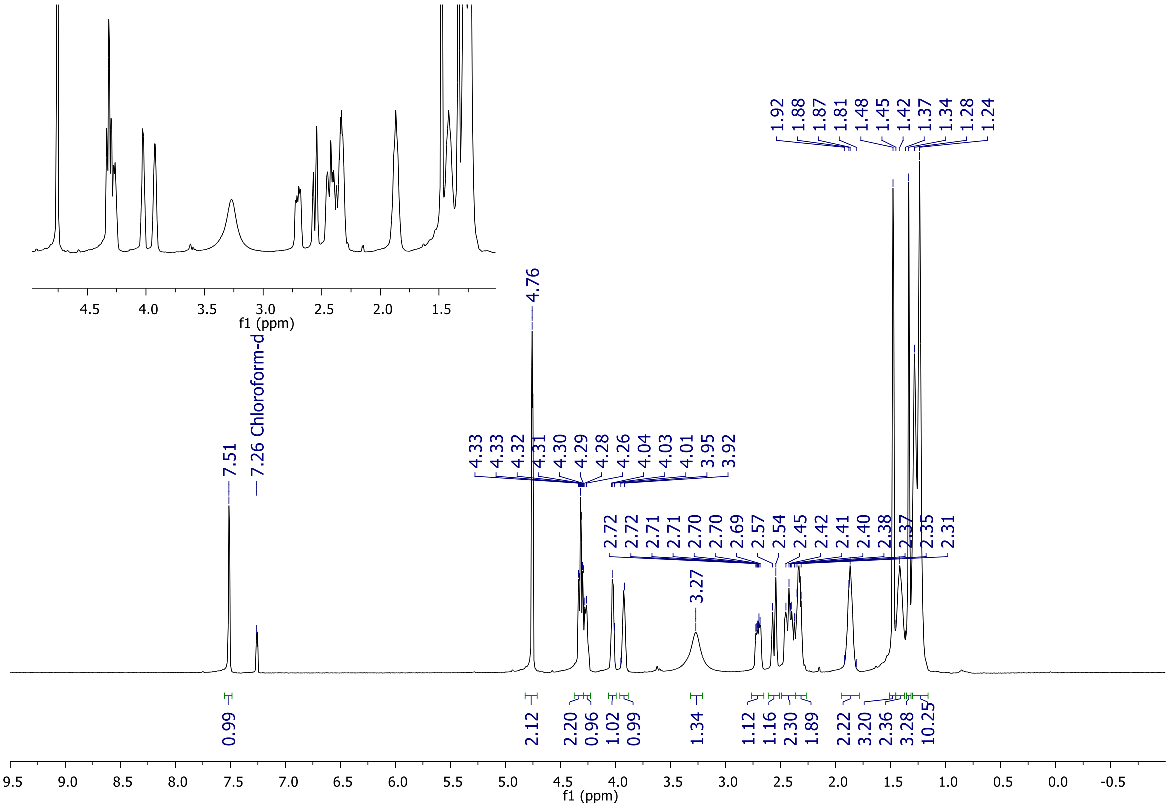

Figure S15: ^{1}H-NMR spectrum of compound **17** (400 MHz, $CDCl_3$)

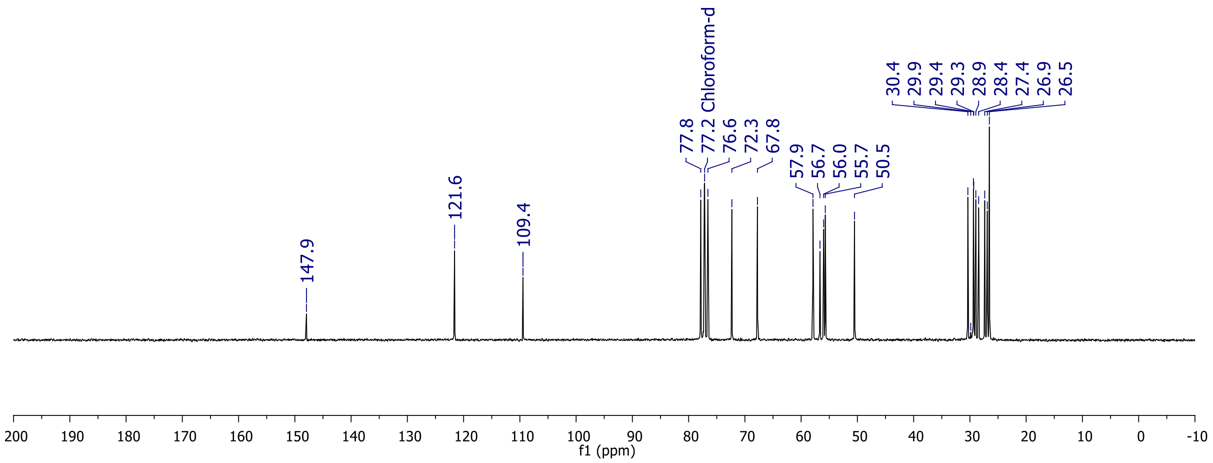

Figure S16: ^{13}C-NMR spectrum of compound **17** (50 MHz, $CDCl_3$).

Synthesis of compound 18: To a solution of **17** (73 mg, 0.18 mmol) in 8 mL of methanol, 150 µL of 37% HCl were added and the mixture was stirred at room temperature for 18 hours. After that a TLC analysis (CH_2Cl_2:MeOH 10:1) showed disappearance of the starting material (*R*f = 0.59), the solvent was removed under reduced pressure. The product, obtained as its hydrochloric salt, was then treated with the strongly basic resin Ambersep 900-OH to afford the final product **18** (62 mg, 0.18 mmol, 95% yield) as a colourless oil. $[\alpha]_D^{21}$= -18.0 (c = 0.94 in CH_3OH); ^{1}H-NMR (400 MHz, CD_3OD): δ= 7.90 (s, 1H, H triazole), 4.68 (s, 2H, C*H₂*OH), 4.39 (t, *J* = 6.4 Hz, 2H, H-9'), 3.90-3.89 (m, 1H, H-3), 3.81-3.77 (m, 1H, H-5), 3.40-3.39 (m, 1H, H-4), 2.80-2.69 (m, 2H, Ha-6, Ha-2), 2.37-2.32 (m, 2H, H-1'), 2.26 (d, *J* = 11.0 Hz, 1H, Hb-2), 2.14-1.99 (m, 1H, Hb-6), 1.91-1.88 (m, 2H, H-8'), 1.51-1.48 (m, 2H, H-2'), 1.31 (bs, 10H, from H-3' to H-7') ppm; ^{13}C-NMR (50 MHz, CD_3OD): δ= 149.0 (*C*-CH_2OH), 124.0 (*C*H triazole), 75.3 (C-4), 69.5 (C-5), 69.1 (C-3), 59.3 (C-1'), 58.2 (C-2), 57.7 (C-6), 56.5 (*C*H_2OH), 51.3 (C-9'), 31.3 (C-8'), 30.5-27.4 (6C, from C-2' to C-7') ppm; MS (ESI): *m/z* calcd (%) for $C_{17}H_{32}N_4O_4$ 356.24; found: 379.52 (43%, $[M+ Na]^+$), 357.52 (100%, $[M+ H]^+$). Elemental analysis: $C_{17}H_{32}N_4O_4$ (356.47) calcd. C, 57.28; H, 9.05; N, 15.72; found C, 57.35; H, 8.99, N, 15.59.

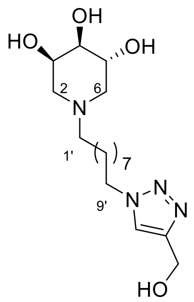

18

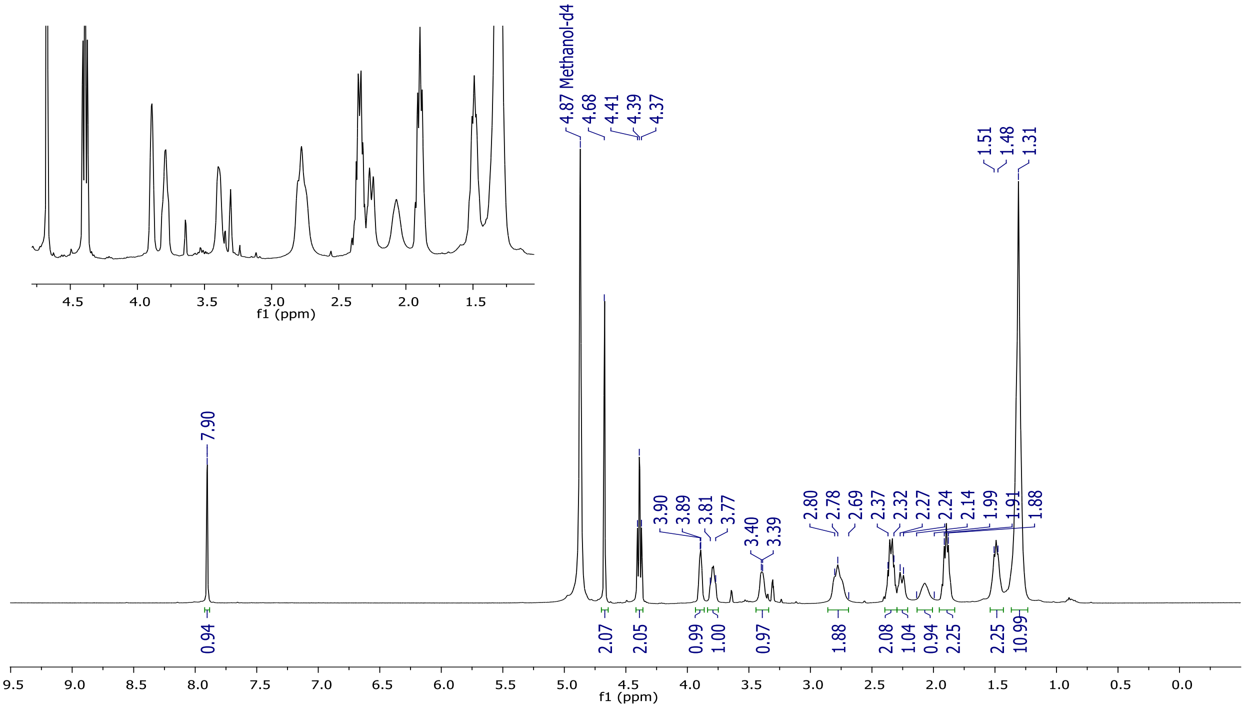

Figure S17: ^{1}H-NMR spectrum of compound **18** (400 MHz, CD_3OD).

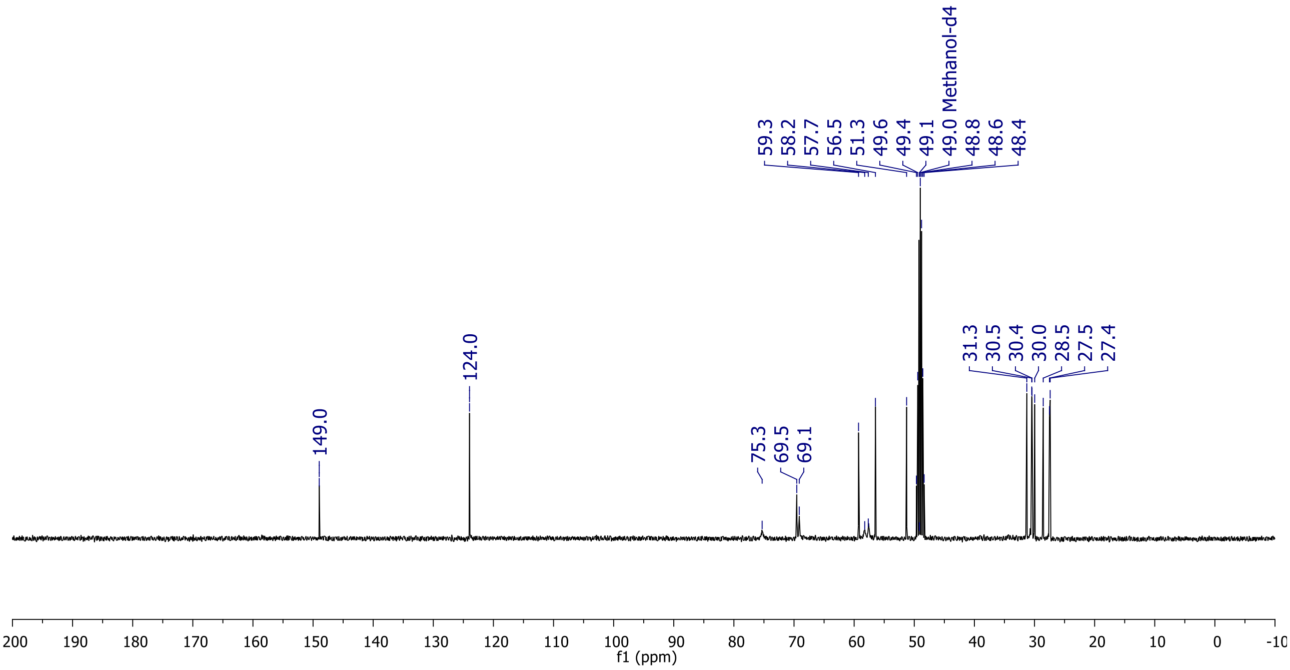

Figure S18: ^{13}C-NMR spectrum of compound **18** (50 MHz, CD_3OD).

Enzymatic Assays

Inhibitory activity towards human GCase: the compounds **11-15** and **18** were screened towards GCase from leukocytes isolated from healthy donors (controls). Isolated leukocytes were disrupted by sonication, and a micro BCA protein assay kit (Sigma–Aldrich) was used to determine the total protein amount for the enzymatic assay, according to the manufacturer instructions. Enzyme activity was measured in a flat-bottomed 96-well plate. Compound solution (3 µL), 4.29 µg/µL leukocytes homogenate (7 µL), and substrate 4-methylumbelliferyl-β-D-glucoside (3.33 mM, 20 µL, Sigma–Aldrich) in citrate/phosphate buffer (0.1:0.2, M/M, pH 5.8) containing sodium taurocholate (0.3%) and Triton X-100 (0.15%) at 37 °C were incubated for 1 h. The reaction was stopped by addition of sodium carbonate (200 µL; 0.5M, pH 10.7) containing Triton X-100 (0.0025 %), and the fluorescence of 4-methylumbelliferone released by β-glucosidase activity was measured in SpectraMax M2 microplate reader (λex=365 nm, λem=435 nm; Molecular Devices). Percentage GCase inhibition is given with respect to the control (without iminosugar). Data are mean ± SD (n=3).

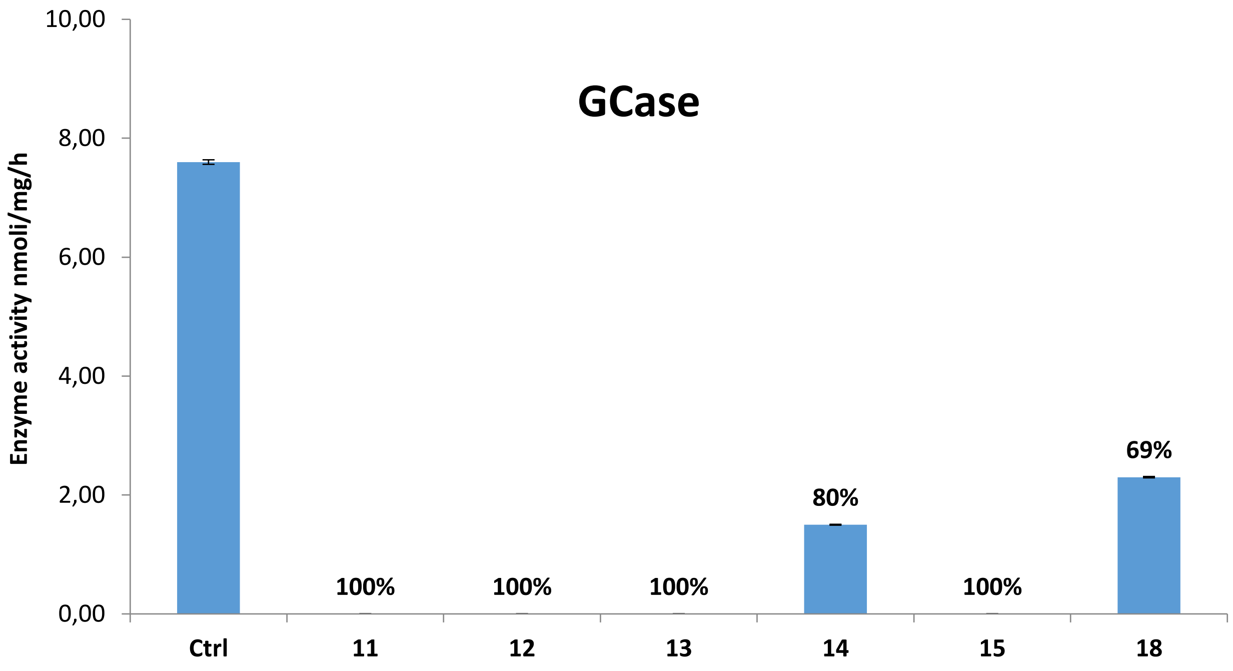

Figure S19: Activity of GCase in the presence of compounds (1 mM). The corresponding calculated percentage of inhibition is indicated above each bar.

IC_{50} determination: The IC_{50} values of inhibitors against GCase were determined by measuring the initial hydrolysis rate with 4-methylumbelliferyl-β-D-glucoside (3.33 mM). Data are mean ± SD (n=3). Data obtained were fitted to the following equation using the Origin Microcal program.

$$\frac{Vi}{Vo} = \frac{Max - Min}{1 + \left(\frac{x}{IC_{50}}\right)^{slope}} + Min$$

where V_i/V_o, represent the ratio between the activity measured in the presence of the inhibitor (V_i) and the activity of the control without the inhibitor (V_0), "x" the inhibitor concentration, Max and Min, the maximal and minimal enzymatic activity observed, respectively.

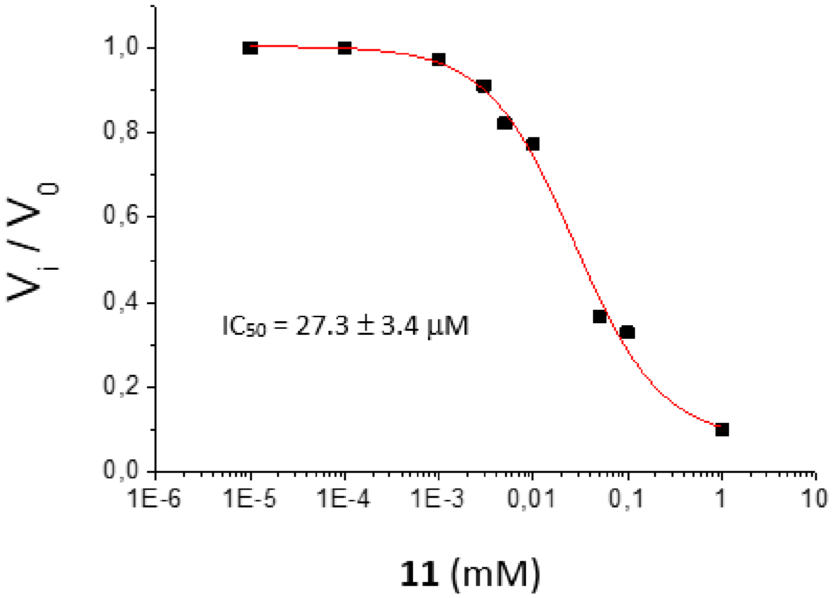

Figure S20: IC_{50} of compound **11** towards GCase from leukocytes isolated from healthy donors.

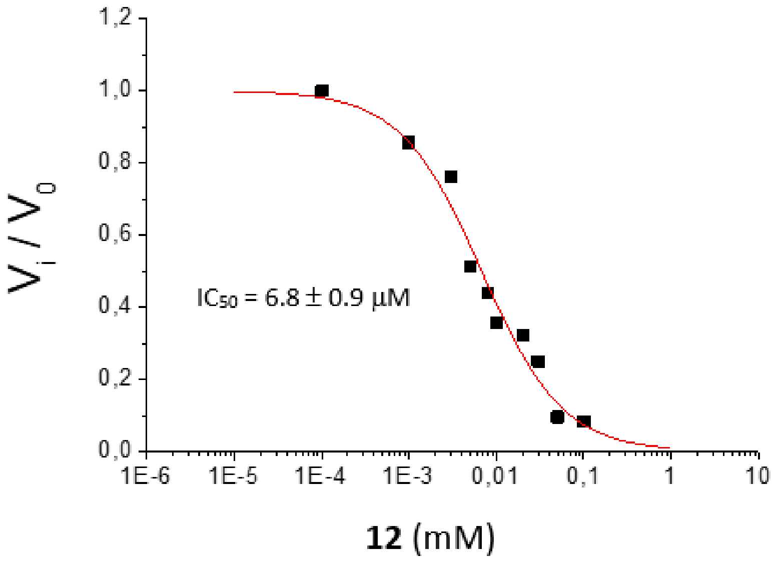

Figure S21: IC_{50} of compound **12** towards GCase from leukocytes isolated from healthy donors.

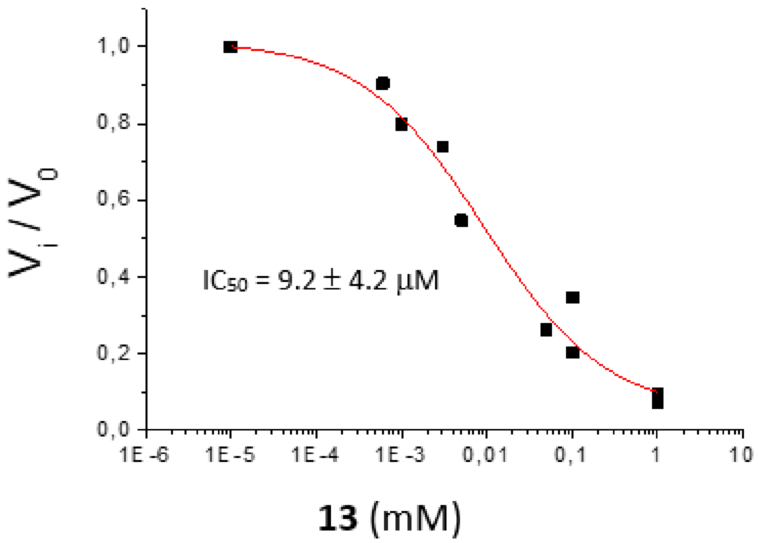

Figure S22: IC_{50} of compound **13** towards GCase from leukocytes isolated from healthy donors.

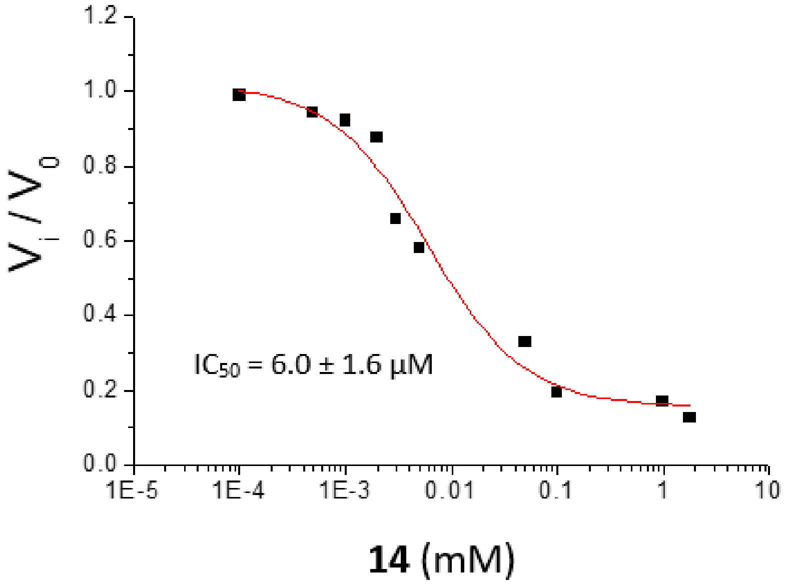

Figure S23: IC_{50} of compound **14** towards GCase from leukocytes isolated from healthy donors.

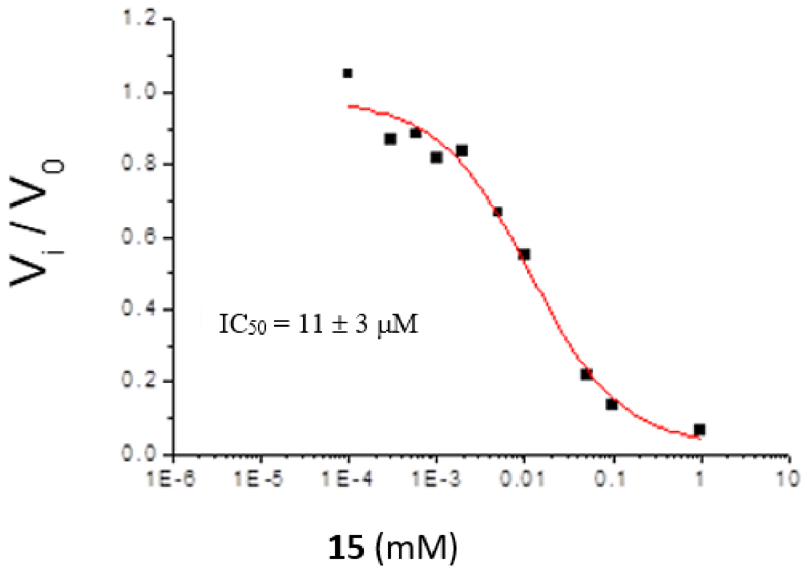

Figure S24: IC_{50} of compound **15** towards GCase from leukocytes isolated from healthy donors.

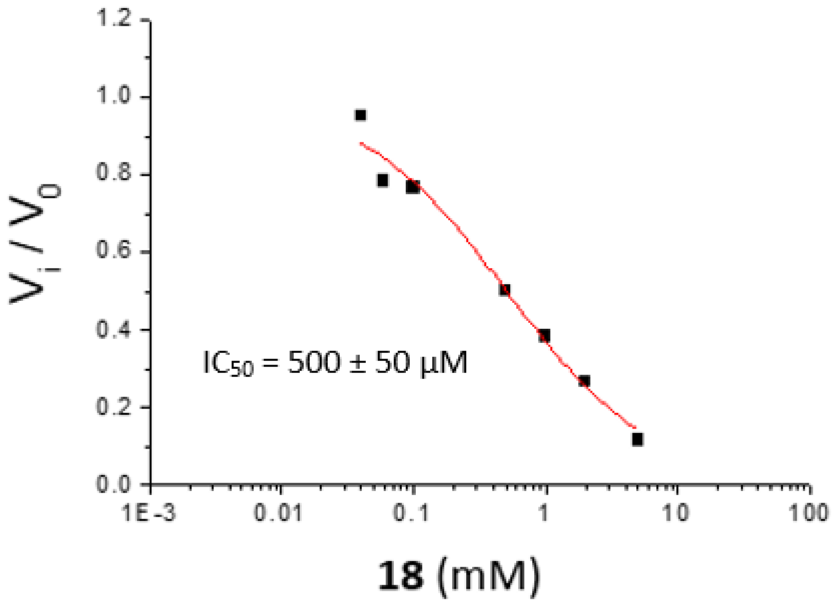

Figure S25: IC_{50} of compound **18** towards GCase from leukocytes isolated from healthy donors.

IC_{50} determination of 12 towards recombinant wild-type human GCase:

The IC_{50} value of **12** against recombinant wild-type human GCase enzyme (VPRIV®, enzyme was diluted in bovine serum albumin (0.2%) at final concentration of 1.0 x 10^{-9} mg/mL) were determined by measuring the initial hydrolysis rate with 4-methylumbelliferyl-β-D-glucoside at different concentrations of **12**.
A proper solution of **12** (3 µL), recombinant wild-type human GCase enzyme solution (7 µL) and substrate 4-methylumbelliferyl-β-D-glucoside (3.33 mM, 20 µL, Sigma–Aldrich) in citrate/phosphate buffer (0.1:0.2, M/M, pH 5.8) containing sodium taurocholate (0.3%) and Triton X-100 (0.15%) at 37 °C were incubated for 1 h. The reaction was stopped by addition of sodium carbonate (200 µL; 0.5M, pH 10.7) containing Triton X-100 (0.0025 %), and the fluorescence of 4-methylumbelliferone released by β-glucosidase activity was measured in SpectraMax M2 microplate reader (λex=365 nm, λem=435 nm; Molecular Devices). Percentage GCase inhibition is given with respect to the control (without iminosugar). Data are mean ± SD (n=3). Data obtained were fitted accordingly to the above-mentioned equation and using the Origin Microcal program.

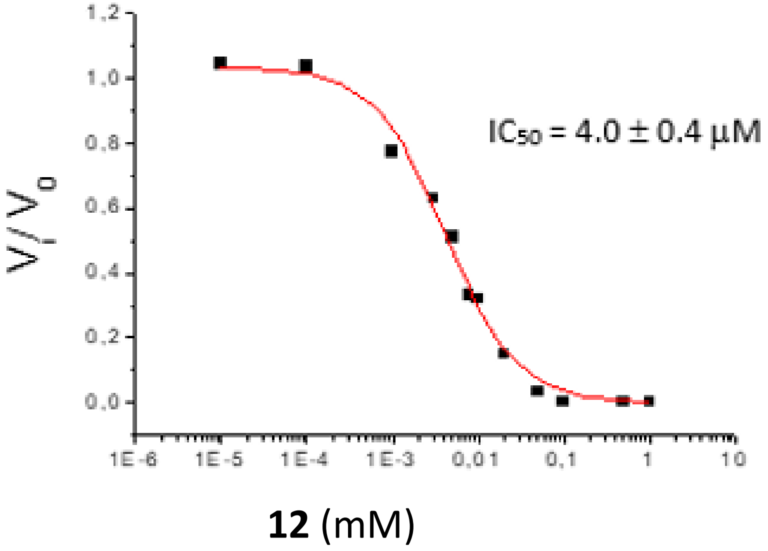

Figure S26: IC_{50} of compound **12** towards recombinant GCase (VPRIV®).

K*i* determination of 12

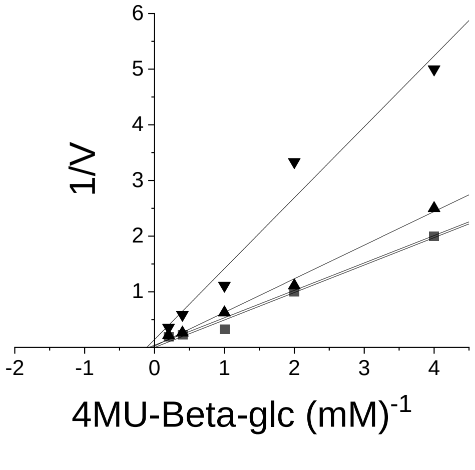

Figure S27: Lineweawer-Burk plot obtained using increasing concentration of compound **12**. The concentrations used were: 0 µM (■), 0.05 µM (○), 0.5 µM (▲), 5 µM (▼).

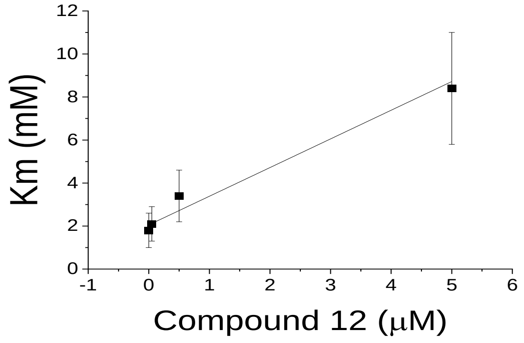

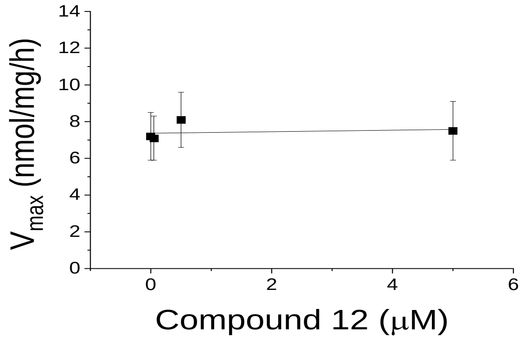

Figure S28: Dependence of Km and Vmax from the concentration of compound **12**. The Km and Vmax were calculated measuring the initial hydrolysis rates in the presence of increasing substrate concentrations (0, 0.05, 0.5, and 5.0 µM) and fitting data using the Michaelis-Menten equation. Fitting of the data was carried out using the nonlinear curve fitting software Origin Pro 2021 (OriginLab Corporation, Massachusetts, USA). Data showed in the figure represent the value ± SE.

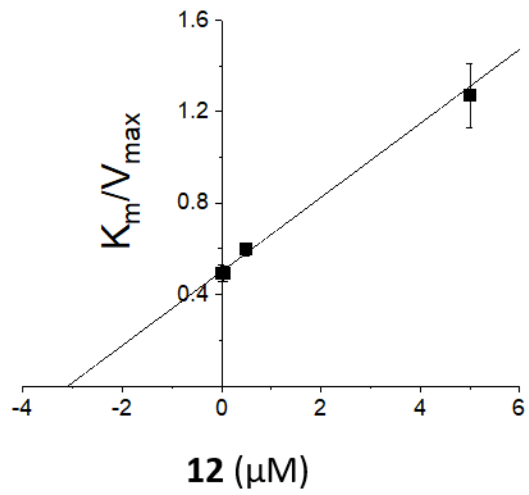

Figure S29. Secondary plot: Km/Vmax versus the concentration of compound **12**. The inhibition constant (K*i*) was determined using the appropriate equation. The calculated K*i* value was 3.1 ± 0.2 μM.

The mechanism of action of compound **12** was determined by studying the dependence of the main kinetic parameters (Km and Vmax) on the increase in inhibitor concentration. We observed that the Km value increased with increasing inhibitor concentration, while the Vmax did not change (Figure S28). Furthermore, the double reciprocal plot showed that the experimental points described straight lines intersecting each other at a point on the ordinate axis (Figure S27). Together, these data suggested that compound **12** behaves as a pure competitive inhibitor with respect to GCase. Therefore, using the appropriate equation, we calculated the value of the inhibition constant (K*i*) which was 3.1 ± 0.2 μM (Figure S29).

Chaperoning activity assays

Evaluation of the effect of multimeric iminosugars (**12, 13,15**) **on GCase activity in Gaucher patients' cells:** Fibroblasts with the N370S/RecNcil (or L444P/L444P) mutation from Gaucher disease patients were obtained from the "Cell line and DNA Biobank from patients affected by Genetic Diseases" (Gaslini Hospital, Genova, Italy). Fibroblasts cells (20 x10^4) were seeded in T25 flasks with DMEM supplemented with fetal bovine serum (10 %), penicillin/streptomycin (1%), and glutamine (1%) and incubated at 37 °C with 5% CO_2 for 24 h. The medium was removed, and fresh medium containing the multimeric iminosugars was added to the cells and left for 4 days. The medium was removed, and the cells were washed with PBS and detached with tripsin to obtain cell pellets, which were washed four times with PBS, frozen and lysed by sonication in water. Enzyme activity was measured as reported above. Reported data are mean ± S.D. (n=2).

GCase activity in human fibroblasts derived from GD patients bearing N370/RecNcil mutations, measured after four days of incubation.

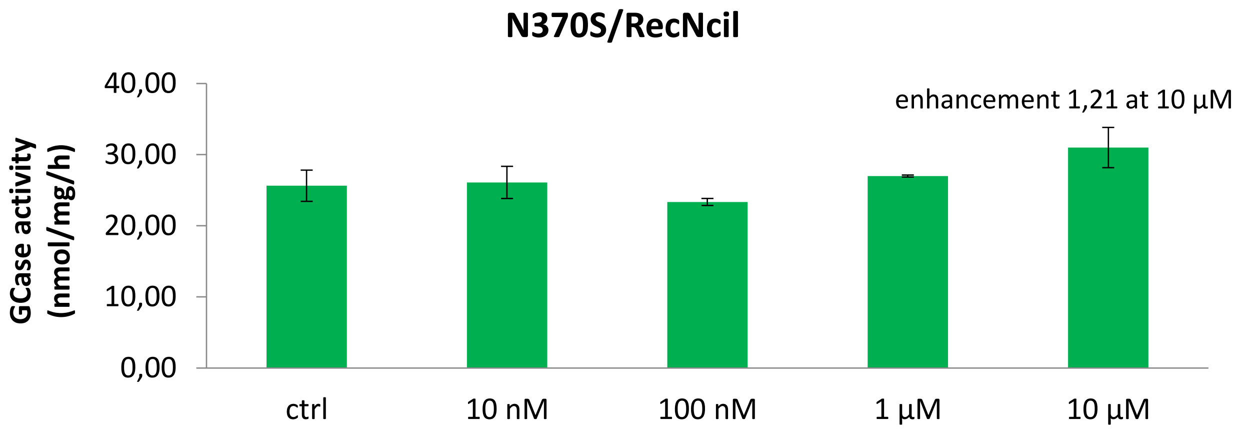

Figure S30: Fibroblasts derived from GD patients bearing N370/RecNcil mutations were incubated without (control, ctrl) or with 6 different concentrations (10 nM, 100 nM, 1 µM, 10 µM, 50 µM, 100 µM) of compound **12**. After 4 days, the flasks containing cells incubated with 100 µM and 50 µM concentrations of **12** showed low cell viability that hampered to proceed with the assay. For the other concentrations, the GCase activity was determined (as above described) in lysates from treated fibroblasts.

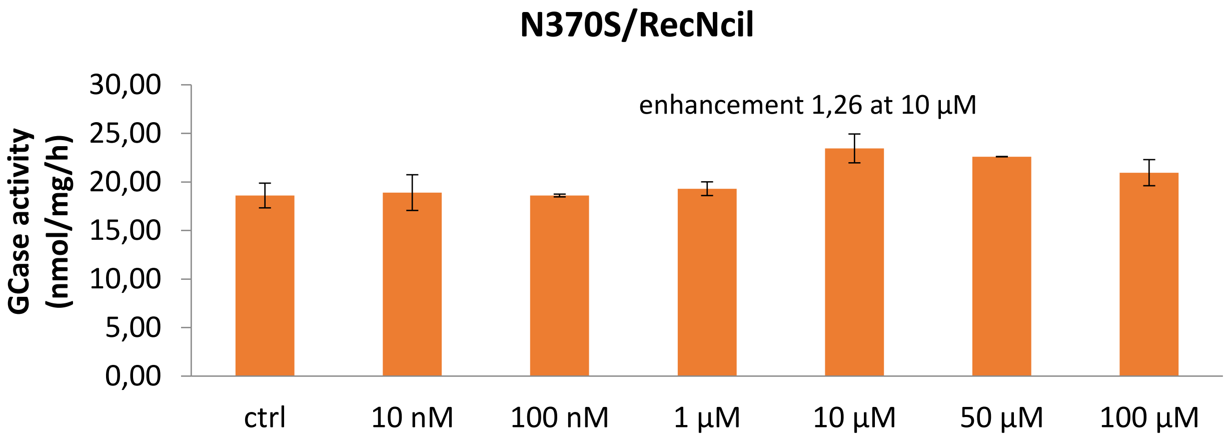

Figure S31: Fibroblasts derived from GD patients bearing N370/RecNcil mutations were incubated without (control, ctrl) or with 6 different concentrations (10 nM, 100 nM, 1 µM, 10 µM, 50 µM, 100 µM) of compound **13**. After 4 days, the GCase activity was determined (as above described) in lysates from treated fibroblasts.

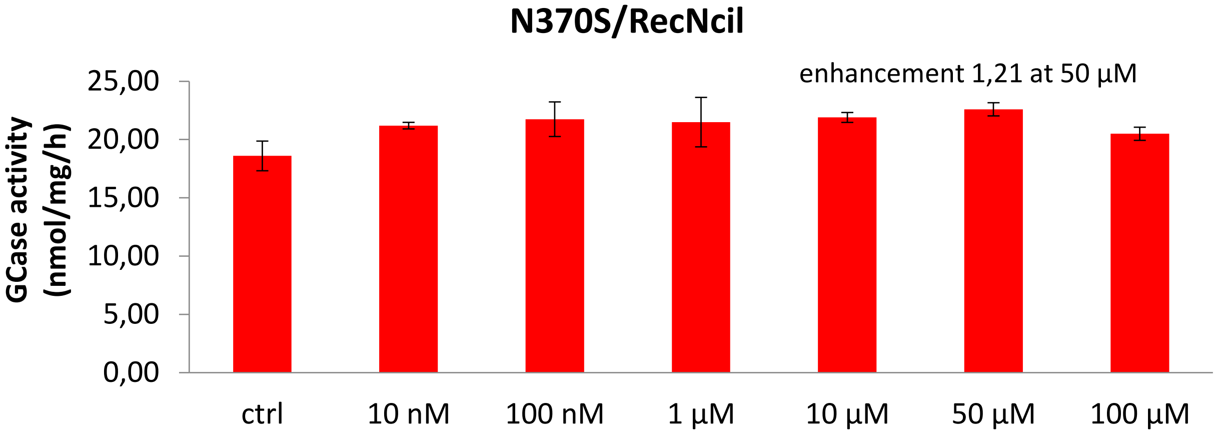

Figure S32: Fibroblasts derived from GD patients bearing N370/RecNcil mutations were incubated without (control, ctrl) or with 6 different concentrations (10 nM, 100 nM, 1 µM, 10 µM, 50 µM, 100 µM) of compound **15**. After 4 days, the GCase activity was determined (as above described) in lysates from treated fibroblasts.

GCase activity in human fibroblasts derived from GD patients bearing L444P/L444P mutations, measured after four days of incubation.

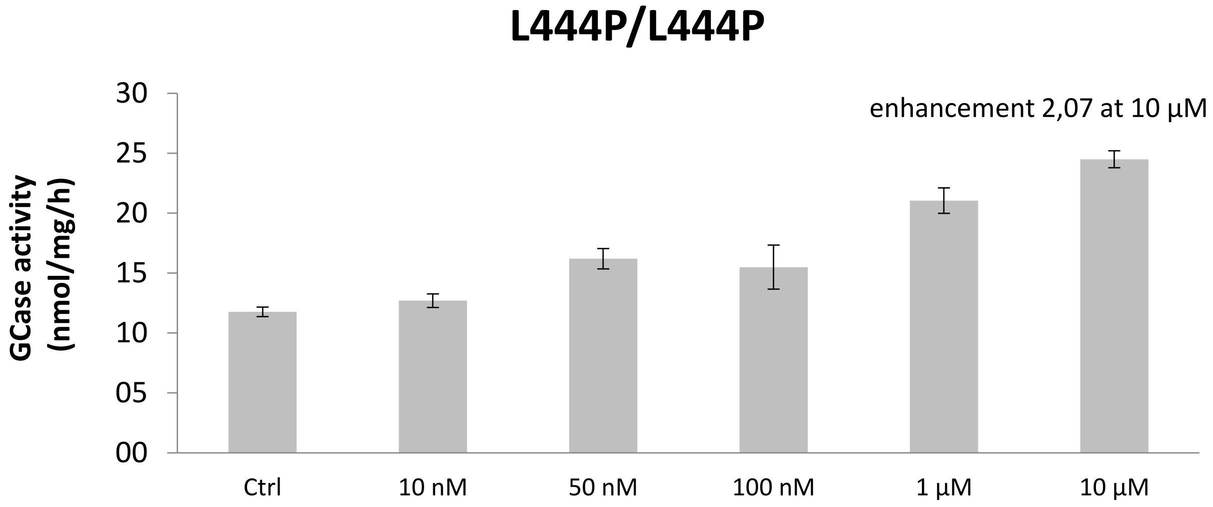

Figure S33: Fibroblasts derived from GD patients bearing L444P/L444P mutations were incubated without (control, ctrl) or with 6 different concentrations (10 nM, 50 nM, 100 nM, 1 µM, 10 µM, 50 µM) of compound **12**. After 4 days, the flasks containing cells incubated with 50 µM concentrations of **12** showed low cell viability that hampered to proceed with the assay. For the other concentrations, the GCase activity was determined (as above described) in lysates from treated fibroblasts.

Thermal Stabilization Assay:[1]

Recombinant wild-type human GCase enzyme (VPRIV® 1.0 x 10^{-9} mg/mL) aliquots (100 µL) with 0 (control), 1, 10, 100, 200 µM of compound **12** were incubated at pH 7.0 for 20 minutes at 0 °C and then for 0 minutes or 20 minutes or 40 minutes or 60 minutes at 48 °C.

Subsequently, 100 µL of water were added in each aliquot. Then each 10 µL aliquot was incubated with 20 µL of substrate 4-methylumbelliferyl-β-D-glucoside (3.33 mM, Sigma–Aldrich) in citrate/phosphate buffer (0.1:0.2, M/M, pH 5.8) containing sodium taurocholate (0.3%) and Triton X-100 (0.15%) at 37 °C, for 1 h. The reaction was stopped by addition of sodium carbonate (200 µL; 0.5M, pH 10.7) containing Triton X-100 (0.0025 %), and the fluorescence of 4-methylumbelliferone released by β-glucosidase activity was measured in SpectraMax M2 microplate reader (λex=365 nm, λem=435 nm; Molecular Devices). Data are mean SD ± (n=3).

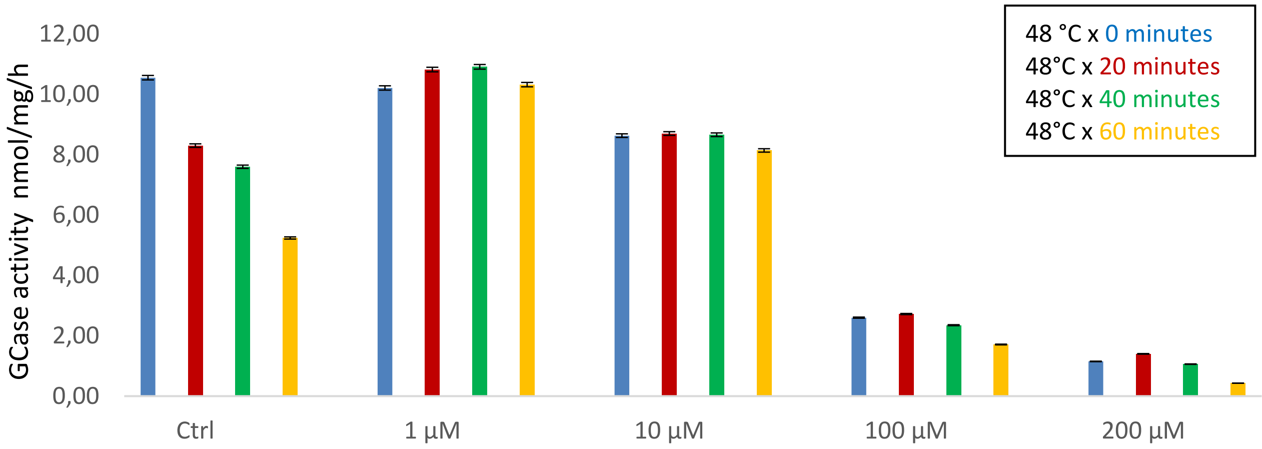

Figure S34: Activity of recombinant human GCase enzyme was determined after incubation for 0 minutes or 20 minutes or 40 minutes or 60 minutes at 48 °C with or without (Ctrl) different concentrations of **12** by measuring the hydrolysis rate with 4-methylumbelliferyl-β-D-glucoside.

[1] L. Dìaz, J. Bujons, J. Casas, A. Llebaria, A. Delgado*, J. Med. Chem*. **2010**, *53*, 5248–5255.
